# A suite of kinetically superior AEP ligases can cyclise an intrinsically disordered protein

**DOI:** 10.1038/s41598-019-47273-7

**Published:** 2019-07-25

**Authors:** Karen S. Harris, Rosemary F. Guarino, Ravindu S. Dissanayake, Pedro Quimbar, Owen C. McCorkelle, Simon Poon, Quentin Kaas, Thomas Durek, Edward K. Gilding, Mark A. Jackson, David J. Craik, Nicole L. van der Weerden, Robin F. Anders, Marilyn A. Anderson

**Affiliations:** 1 La Trobe Institute for Molecular Science, Melbourne, Vic. 3086 Australia; 2 Institute for Molecular Bioscience, The University of Queensland, Brisbane, QLD 4072 Australia

**Keywords:** Ligases, Molecular engineering

## Abstract

Asparaginyl endopeptidases (AEPs) are a class of enzymes commonly associated with proteolysis in the maturation of seed storage proteins. However, a subset of AEPs work preferentially as peptide ligases, coupling release of a leaving group to formation of a new peptide bond. These “ligase-type” AEPs require only short recognition motifs to ligate a range of targets, making them useful tools in peptide and protein engineering for cyclisation of peptides or ligation of separate peptides into larger products. Here we report the recombinant expression, ligase activity and cyclisation kinetics of three new AEPs from the cyclotide producing plant *Oldenlandia affinis* with superior kinetics to the prototypical recombinant AEP ligase OaAEP1_b_. These AEPs work preferentially as ligases at both acidic and neutral pH and we term them “canonical AEP ligases” to distinguish them from other AEPs where activity preferences shift according to pH. We show that these ligases intrinsically favour ligation over hydrolysis, are highly efficient at cyclising two unrelated peptides and are compatible with organic co-solvents. Finally, we demonstrate the broad scope of recombinant AEPs in biotechnology by the backbone cyclisation of an intrinsically disordered protein, the 25 kDa malarial vaccine candidate *Plasmodium falciparum* merozoite surface protein 2 (MSP2).

## Introduction

Proteases are widespread throughout nature and typically act to hydrolyse polypeptide chains. Less frequently, proteases also work as ligases (transpeptidases) to create new peptide bonds. Recently, two plant-derived asparaginyl endopeptidases (AEPs, also known as vacuolar processing enzymes and legumains) that function primarily as ligases, butelase-1 (or CtAEP1) and OaAEP1_b_, have been identified and characterised *in vitro*^1,2^. Although their primary function *in planta* is likely the biosynthesis of cyclotides, a class of highly stable, backbone-cyclised peptides^3^, these enzymes can also cyclise unrelated peptides and proteins that are not naturally cyclic following the addition of short recognition motifs^1,2^. After enzymatic release of the leaving group, as little as one foreign residue remains in the final product, making AEP ligases attractive tools for peptide modification^1^.

Butelase-1 extracted from the cyclotide-producing plant *Clitoria ternatea* is the most extensively studied AEP ligase and can circularise both peptides and proteins as well as label their N- and C-termini^2,4–10^. A recombinant version of butelase 1 has only recently been produced^11^, but has not been biochemically characterised.

OaAEP1_b_ from the cyclotide-producing plant *Oldenlandia affinis* can be recombinantly expressed in bacteria in its active form^1,12^. When examined on similar cyclotide substrates, the turnover rate (*k*_cat_) of native butelase-1 is around 4-fold faster than recombinant OaAEP1_b_^1,2^. However, these and other^12^ kinetic comparisons of AEP ligases are unreliable because the relative levels of active and inactive enzyme in each preparation were not established, potentially distorting the numbers by over-estimating the active enzyme concentration.

In addition to OaAEP1_b_, *O*. *affinis* produces at least two other AEPs (OaAEP2 and OaAEP3) and transcriptomics data indicate that this number is probably higher^1^. OaAEP3 was assigned as a ligase-type AEP using an *in planta* system for rapid screening of AEP activity^13^, but further biochemical characterisation has not yet been conducted. The rapid functional annotation afforded by this *in planta* screening strategy led to the identification of key sequence polymorphisms that underpin AEP ligase activity and distinguish the ligases from the proteases, at least in this context. This includes 12 sites where the nature of the amino acid is associated with enzyme activity preferences and a region called the “Marker of Ligase Activity” (MLA) that is either deleted or highly hydrophobic in the ligase-type AEPs^14^. The importance of these sequence differences was confirmed by mutagenesis: when OaAEP2 was mutated at these key sites and tested on the same target peptide under the same conditions, the dominant product shifted from hydrolysed to cyclised^14^.

Mechanistically, the way in which AEP ligases favour ligation over hydrolysis remains poorly understood. Like another cyclising enzyme, the serine protease PatG^15^, a cyclisation model in which the C-terminal propeptide remains bound to the active site until displaced by an incoming N-terminus has been proposed for AEPs, with the presence of a P2′ residue (the second residue after the scissile bond) being deemed particularly important^1,16,17^. This is supported by molecular dynamics simulations and structural analysis, which suggest that the nature of the S2′/P2′ interaction is important for ligation^18^.

Recently, the field has been complicated by the identification of four plant AEPs with ligase activity that emerges only as the pH approaches neutrality^18–20^. These AEPs are derived from plant species that are not reported to produce cyclotides (*Helianthus annuus*, *Arabidopsis thaliana* and *Ricinus communis*) and they work as proteases under acidic conditions (pH 5). Although these enzymes have been termed “AEP ligases”, only those from *A*. *thaliana* (AtLEGγ and AtLEGß) are efficient ligases (i.e. produce predominantly cyclic product)^18,19^. The others (HaAEP1 and RcAEP1) are relatively inefficient, at least on the substrate tested, because they produce relatively high amounts of linear, hydrolyzed side-product compared to cyclic product^20^. These findings highlight the need for clarity around how AEP ligases, AEP proteases and dual-function AEPs are defined.

There is currently great interest in the application of AEP ligases in biotechnology and understanding their scope and key requirements for activity will be crucial to realising this potential. It is known that AEP ligases can cyclise a wide range of native and non-native peptide targets after the addition of appropriate AEP recognition motifs and that this does not require a pre-formed peptide structure that brings the N-and C-termini in close proximity; indeed, the kinetics of cyclotide maturation improve when the rigid disulfide-bonded structure maintaining the N- and C-termini in close proximity is eliminated^1,2^. Although examples of *protein* cyclisation are thus far limited to globular proteins with well-defined structures and adjacent N- and C- termini^6^, the peptide findings imply that proteins that fall outside of these parameters could be cyclised if they are sufficiently flexible. Intrinsically disordered proteins meet this brief because they lack a stable three-dimensional structure, either entirely or in specific regions, and exist as a dynamic conformational ensemble^21–23^. This disorder can take the form of extended (random-coil) or collapsed (partially folded) regions making this a heterogeneous group of proteins^22,24,25^. However, the transient juxtaposition of N- and C-termini allowed by their inherent flexibility may permit their cyclisation by AEP ligases.

The 25 kDa malarial vaccine candidate merozoite surface protein 2 (MSP2) is intrinsically disordered when produced recombinantly^26,27^, but the native antigen is more ordered, and antigenically distinct from the recombinant protein^28^, creating a challenge for vaccine design. Although the precise structure of MSP2 at the parasite surface is not known, native MSP2 may adopt a pseudo-cyclic structure generated by a membrane-bound C-terminal GPI-anchor^29^ and possible association of the N-terminal region with the membrane^27,30,31^. MSP2 was therefore selected as a candidate for AEP-mediated cyclisation for two reasons: firstly, to explore the capabilities of AEP ligases and, secondly, to investigate the hypothesis that backbone-cyclised MSP2 is antigenically similar to the native antigen.

In this study, we report the recombinant expression and characterisation of three new *O*. *affinis* AEPs: OaAEP3, and the newly identified OaAEP4 and OaAEP5. These enzymes are kinetically superior to OaAEP1_b_ and maintain their preferential ligase activity across a broad pH range and in commonly used organic co-solvents when tested on R1 model peptides. These AEP ligases (including OaAEP1_b_) do not shift their activity preferences according to pH, and we therefore propose that they be termed “canonical AEP ligases” to distinguish them from AEPs with ligase activity that is dependent on pH (here termed “pH-dependent ligases”). We use substrate mutagenesis to profile enzyme selectivity and provide mechanistic evidence that canonical AEP ligases intrinsically prefer ligation over hydrolysis. Finally, we use the malarial vaccine candidate MSP2 as an example to show that these AEP ligases can cyclise an intrinsically disordered protein, showcasing the broad utility of canonical AEP ligases.

## Results

### Identification and recombinant expression of a family of recombinant peptide ligases from *O*. *affinis*

**Figure 1:**
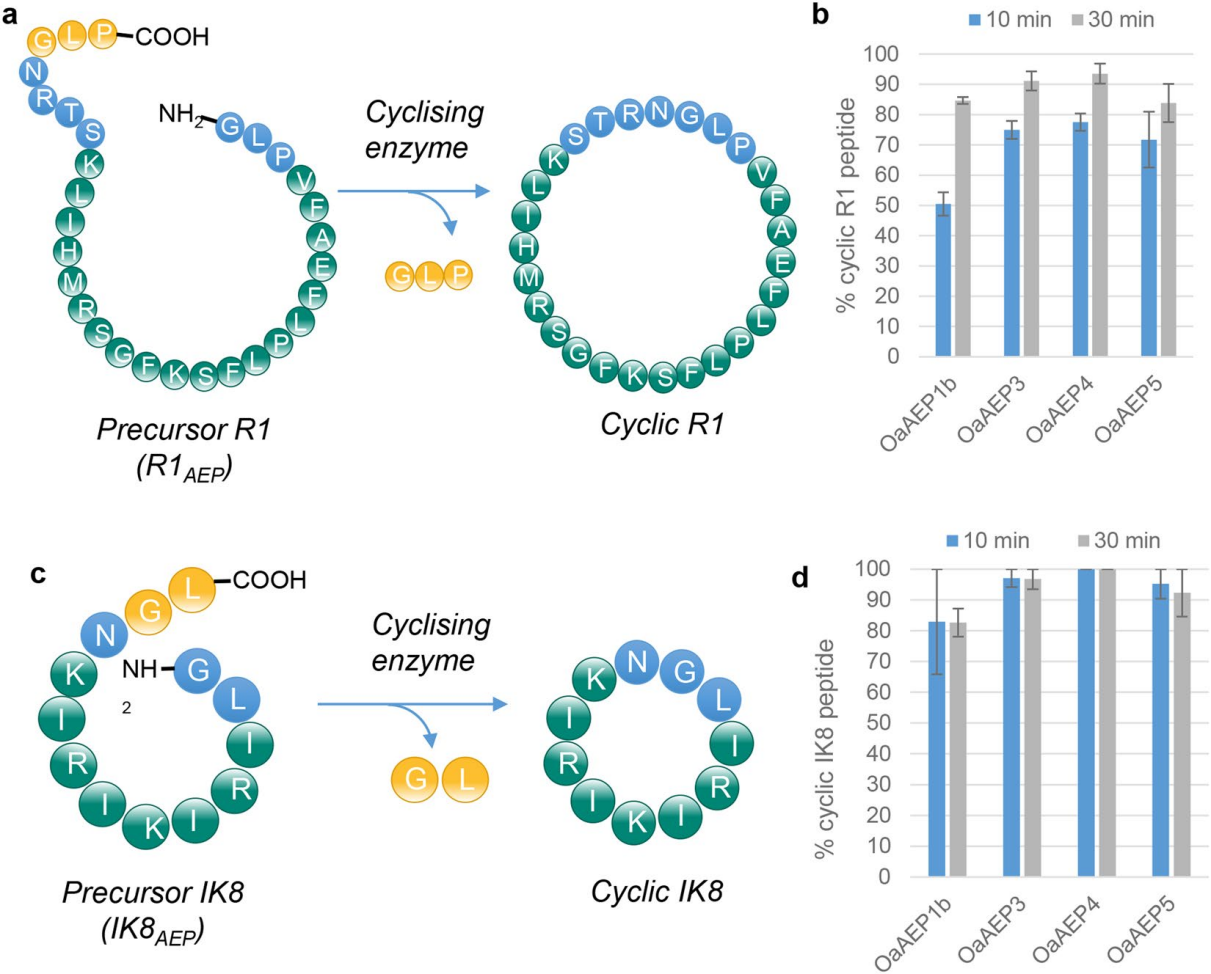
*Recombinant enzymes rapidly and efficiently cyclise non-native substrates*. Recombinant AEPs were incubated with the non-native substrates (**a**) R1_AEP_ or (**c)** IK8_AEP_ peptide (each at 280 µM) and the products were assessed by MALDI-MS. The proportion of cyclic product was determined relative to all peaks attributed to the processed or unprocessed (**b**) R1 peptide or (**d**) IK8c peptide. Enzyme concentrations OaAEP1_b_ 0.528 µM, OaAEP3 0.132 µM, OaAEP4 0.185 µM, OaAEP5 0.132 µM. The concentration of OaAEP1_b_ was significantly higher as lower concentrations gave poor levels of conversion to cyclic product in the time frames tested. The assays were conducted in activity buffer (50 mM sodium acetate buffer, pH 5.0, 0.5 mM NaCl, 1 mM EDTA, 0.5 mM TCEP) at room temperature. The bar charts show mean values where n = 3 ± SEM. Reactions were stopped by heating at 70 °C for 5 min.

To enable *in vitro* biochemical characterisation of the AEP ligase OaAEP3, it was expressed recombinantly in *E*.*coli*, along with two new AEPs, OaAEP4 and OaAEP5, and the previously described OaAEP1_b_ (Fig S1). OaAEP4 and OaAEP5 were derived from transcriptomics analysis of *O*. *affinis*, but their existence remained theoretical as they could not be amplified from a cDNA library^1^ and for this study were obtained as synthetic genes. OaAEP4 and OaAEP5 were predicted to be ligases based on the presence of a ligase-type MLA^14^. The sequences of the four AEPs studied here (OaAEP1_b_, OaAEP3, OaAEP4 and OaAEP5) were >81% identical at the protein level as determined by the Clustal Omega multiple alignment tool (Table S1). All recombinant enzymes were confirmed as efficient ligases when assayed on two different target peptides carrying AEP recognition motifs: the eight residue anti-microbial peptide IK8 (IK8_AEP_)^32^ and the 20 residue anti-malarial peptide R1 (R1_AEP_)^33^ (Fig. 1). Yields approaching 100% cyclic product could be achieved within 10 min with as little as 0.0005 nmol of enzyme per nmol of substrate (i.e. a 0.0005 enzyme:substrate molar ratio).

### Real-time kinetic characterisation of recombinant AEP ligases

Direct comparison of the kinetic parameters previously reported for AEP ligases is unreliable because the concentration of active enzyme was not determined^1,2,12^. In enzyme kinetics, it is important to distinguish the active enzyme component from denatured enzyme and other contaminants and this can be achieved by titration of the active site with an inhibitor^34^. The reversible caspase inhibitor Ac-YVAD-CHO has been reported to be an AEP inhibitor^35^ but this compound is poorly effective on OaAEP1_b_ and not appropriate for active site titration^1^. In this study, we used the irreversible caspase inhibitor Ac-YVAD-CMK as an active site titrant to determine the active concentration of each recombinant enzyme, enabling more accurate comparison of kinetic parameters (Supplementary Fig. S2; Table 2).

To avoid the cumbersome discontinuous kinetic assays used previously^1,2,12^, we designed a cyclisable internally-quenched fluorescent (IQF) peptide substrate based on the R1 peptide (R1_IQF_) so that product formation could be tracked in real time. The major processing product of the R1_IQF_ substrate was confirmed as cyclic peptide by MALDI-MS (Supplementary Fig. S3). A minor peak consistent with linear product (+18 Da from the cyclic peak) was also observed (Supplementary Fig. S3), but the proportion of this was negligible relative to total product. This confirmed that this assay is a reliable measure of cyclisation kinetics.

Michaelis-Menten kinetics using R1_IQF_ revealed that the turnover rates (*k*_cat_) of all four enzymes were remarkably similar: 0.59–0.99 s^−1^ (Table 1; Supplementary Fig. S4). However, dramatically different *K*_m_ values led to large differences in catalytic efficiencies. The most efficient enzyme on the R1_IQF_ substrate was OaAEP3 with a *k*_cat_/*K*_m_ value of 329,982 M^−1^s^−1^. Interestingly, OaAEP1_b_ had the highest turnover rate (*k*_cat_ 0.99 s^−1^) but was the poorest performing enzyme in the initial cyclisation assays, requiring approximately 4-fold more enzyme to achieve similar outcomes (Fig. 1). This is likely to be due to an over-estimation of the maximum enzyme velocity because the apparent *K*_m_ (146 µM) was much higher than the substrate concentration that could feasibly be used in this assay (refer to Supplementary Fig. S4). Importantly, OaAEP3, OaAEP4 and OaAEP5 all had much lower *K*_m_ values (<0.77–2.4 µM) for the IQF substrate, indicating that maximum turnover rate (*k*_cat_) could be reached at very low substrate concentrations, suggesting these enzymes have a higher affinity for this substrate.

**Table 1:** Kinetic parameters of IQF peptide processing. Substrate R1_IQF_: GLPVFAEFLPLFSKFGSRMHIL(K-Abz)STRN↓GLPSY(3NO2).

Enzyme	*k*cat (s^−1^) ±sem	*K*_m_ (µM) ±sem	k_cat_/*K*_m_ (M^−1^s^−1^)
OaAEP1_b_	0.99 ± 0.06	146 ± 16	6774
OaAEP3	0.61 ± 0.07	1.86 ± 0.8	329,982
OaAEP4	~0.76	<0.77	~983,865
OaAEP5	0.59 ± 0.01	2.4 ± 0.43	247,117

### Substrate specificity of recombinant AEP ligases

**Figure 2:**
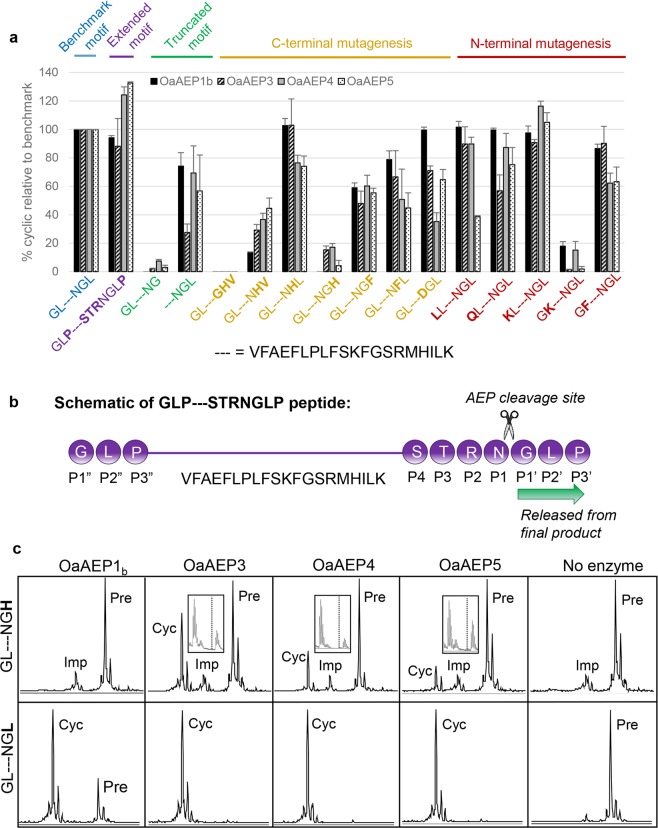
*Specificity of recombinant AEPs*. (**a**) The ability of recombinant AEPs to cyclise the R1 peptide (represented by–) with various flanking sequences was determined. The proportion of cyclic product is displayed relative to the benchmark motif (GL–NGL). The enzymes were added at a nominal concentration of 19.7 µg mL^−1^ based on quantitation by BCA assay. Incubation times were such that the proportion of cyclic product from the peptide carrying the benchmark motif (GL-NGL) was between 72–81% (OaAEP1_b_ and OaAEP5, 1 h; OaAEP3, 35 min; OaAEP4, 5 h). The assays were performed in activity buffer (50 mM sodium acetate buffer, pH 5.0, 50 mM NaCl, 1 mM EDTA, 0.5 mM TCEP) at room temperature. The chart shows mean values where n ≥ 3 ± SEM. (**b**) Schematic describing the nomenclature of N- and C-terminal AEP recognition motifs. The peptide with the extended motif (GLP–STRNGLP) is used as an example (see also Fig. 1a). All residues downstream of the P1 site are released from the final product. (**c**) MALDI MS spectra of the GL–NGL and GL–NGH peptides 5 hours post-enzyme addition (0.132 µM enzyme, pH 5). Boxed inset zooms in on the region containing the processing product. The first peak is cyclic product and the second peak is likely a + 22 Da sodium adduct. The expected position of linear processing product (monoisotopic mass) is shown by the dashed line.

*O*. *affinis* contains at least 17 different cyclotides^36^ and thus it is likely that the plant produces AEPs with different specificities. To compare the preferred recognition motifs of the four recombinant AEP ligases, activity against a panel of R1 peptides carrying varied flanking residues was determined relative to a “benchmark” recognition motif (GL–NGL, where–represents the R1 peptide) (Fig. 2a,b), under conditions where the benchmark cyclic peptide yield was between 72 and 81%. As reported for OaAEP1_b_^1^, NGL was the minimal C-terminal recognition motif for all enzymes, since further truncation reduced the yield to less than 10% of the benchmark.

Overall, the enzymes displayed a similar pattern of sequence requirements, reflecting their high level of identity. However, some subtle differences were observed. For example, OaAEP1_b_ was significantly more tolerant to Asp in the P1 position (GL–**D**GL) when compared with the three other enzymes (OaAEP3 p < 0.03, OaAEP4 p < 0.0001, OaAEP5 p < 0.006, Tukey’s multiple comparisons test), whereas OaAEP4 was superior when Lys was in the P1” position (**K**L–NGL), although this was only significant when compared with OaAEP3 (p < 0.003, Tukey’s multiple comparisons test).

As reported for OaAEP1_b_^1^, the minimum foreign residue footprint in the cyclised product was one residue (refer to R1 variants–NGL and GL–NG). There was some flexibility in the composition of the AEP recognition motifs, but the P2′ and P2″ positions (defined in Fig. 2b) were particularly sensitive to the presence of basic residues (refer to peptides GL–NG**H** and G**K**–NGL), with relative yields falling below 20% of the benchmark.

### Influence of the substrate P2′ residue on AEP ligase activity

The enzyme S2′ binding pocket accommodates the substrate P2′ residue and this interaction has been deemed particularly important for AEP ligase activity^1,17^. In AtAEPγ, the S2′ binding pocket consists of the residues Val^182^, Tyr^192^, Tyr^190^ and Gly^184 ^^18^ and sequence examination revealed that OaAEP1_b_, OaAEP3, OaAEP4 and OaAEP5 have identical residues at the corresponding sites (Supplementary Fig. S5a). To assess if the ligase activity of our recombinant *O*. *affinis* AEPs also requires a hydrophobic residue in the P2′ position, we compared the products generated from R1 peptides with P2′ residues with either a hydrophobic or charged side chain (GL–NG**L** and GL–NG**H**) (Fig. 2c). Using an extended incubation time compared to those shown in Fig. 2a, OaAEP1_b_ did not process the GL–NG**H** peptide at all, whilst OaAEP3, OaAEP4 and OaAEP5 processed it with far slower kinetics compared to the wild type GL–NG**L** peptide. Importantly, there was no apparent increase in the relative proportion of linear product generated from the GL–NG**H**, indicating that the nature of the P2′ residue influenced the reaction kinetics, but not activity preferences (i.e. ligase versus protease activity) of these AEPs.

### Activity preferences of recombinant *O*. *affinis* AEPs at different pHs

**Figure 3:**
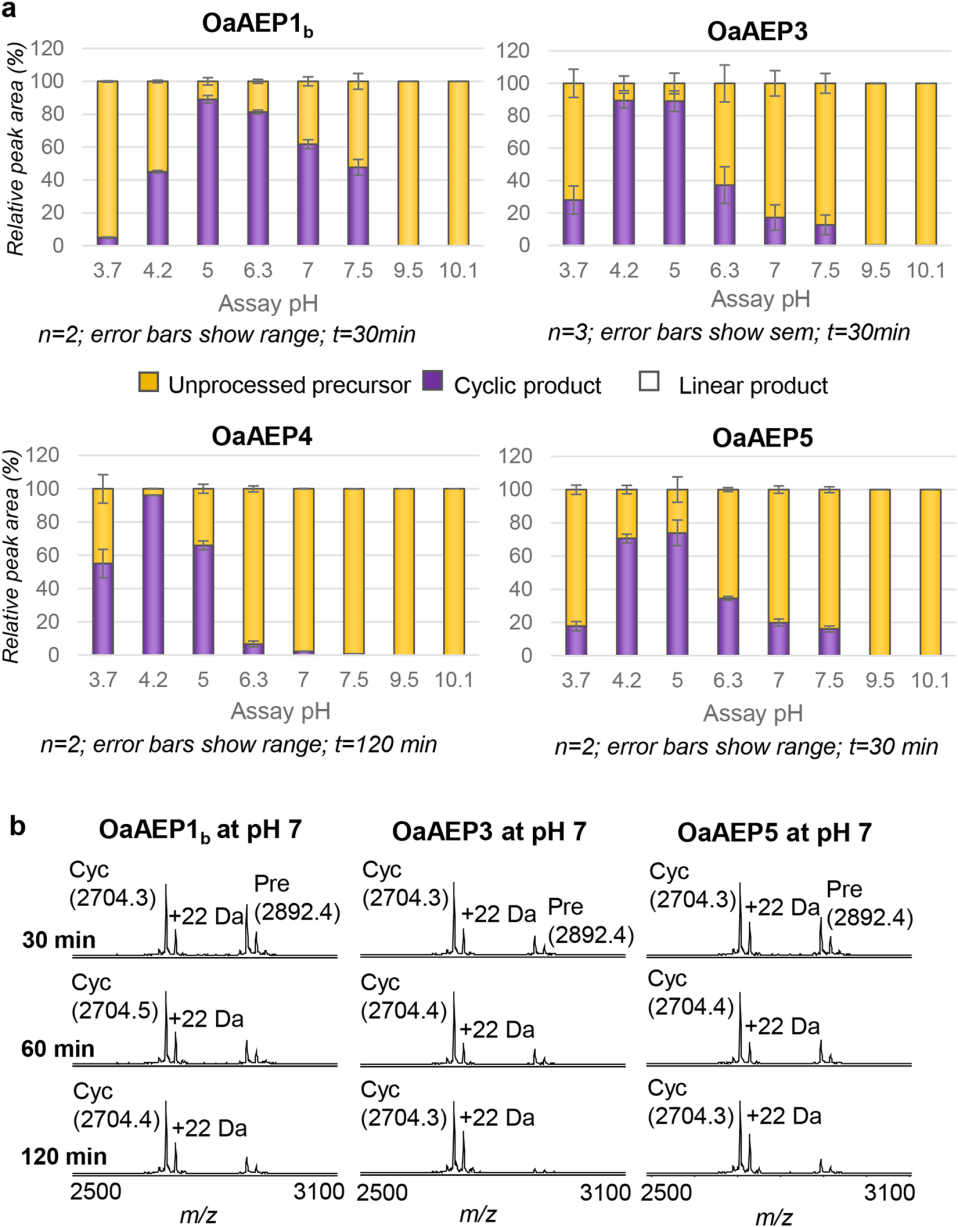
*Impact of pH on activity of recombinant AEP ligases*. (**a**) Recombinant AEPs were incubated with the R1 peptide (GL–NGL variant; 280 µM) at a range of pH values and the products assessed by MALDI-MS. The relative peak area attributable to the cyclic product, linear product or unprocessed precursor was expressed as a % of the total peak area. Enzyme concentrations: OaAEP1_b_ 0.528 µM, OaAEP3 0.132 µM, OaAEP4 0.033 µM, OaAEP5 0.132 µM. OaAEP1_b_ was used at a higher concentration because it was not as active as the other enzymes under the conditions tested. The lower concentration of OaAEP4 reflects the low stock concentration of this enzyme. Reactions were stopped by heating at 70 °C for 5 min. The assay was conducted at room temperature using the same composition as the standard activity buffer (with 50 mM NaCl) but the buffering system was as appropriate for each pH and is detailed in the Materials and Methods. (**b**) Recombinant AEPs were incubated with the R1 peptide (KL–NGL variant; 280 µM) at pH 7 for 30, 60 or 120 min and the products assessed by MALDI-MS. The assays were performed in activity buffer (50 mM phosphate buffer, pH 7.0, 50 mM NaCl, 1 mM EDTA, 0.5 mM TCEP). The proportion of cyclic product was determined relative to all peaks attributed to the processed or unprocessed target peptide. OaAEP1_b_ and OaAEP3 were at 0.528 µM; OaAEP5 was at 0.265 µM. Reactions were stopped by heating at 70 °C for 5 min. The observed monoisotopic masses are listed (Da; [M + H]^+^). The expected monoisotopic mass of cyclic product (cyc) is 3703.5 Da. The expected average mass of precursor (pre) is 2891.6 Da. +22 Da peaks present in precursor and product spectra are likely to represent a sodium adduct. A single representative experiment of two technical replicates is shown.

To determine if our suite of recombinant *O*. *affinis* AEP ligases switch their activity preferences according to pH, we tracked the processing products of the R1 model peptide with optimal N- and C-terminal recognition residues for cyclisation (GL–NGL variant) at different pH values. All enzymes were most active at acidic pH (Fig. 3a) but, in contrast to some other (pH-dependent) ligases, the pH did not impact the activity preferences of recombinant *O*. *affinis* AEPs when tested on this optimal substrate because no linear processing products were observed at any pH (Fig. 3a). We term these AEPs that can continue to work as preferential ligases across a broad pH range “canonical AEP ligases” to distinguish them from other plant AEPs where ligase activity is pH-dependent^19,20^. When the residue composition of these two different types of AEPs (canonical versus pH-dependent AEP ligases) was compared at the 13 ligase predictive sites (including the MLA)^14^ it was evident that only the canonical AEP ligases consistently presented sequence signatures associated with ligase activity (Supplementary Fig. S5b,c).

Ligation assays with OaAEP1_b_ are generally performed at pH 5^1^, but higher pHs will be required for ligation of proteins or peptides that are unstable at acidic pH. Here we show that despite their acidic pH optima, OaAEP1_b_, OaAEP3 and OaAEP5 can also generate excellent yields of cyclic product at pH 7 when increased amounts of enzyme (up to 0.528 µM enzyme with 280 µM substrate) and longer incubation times (up to 2 h) are used (Fig. 3b). Note that the low stock concentration of OaAEP4 precluded the same experiment being carried out for this enzyme.

### Solvent tolerance of recombinant protein ligases

**Figure 4:**
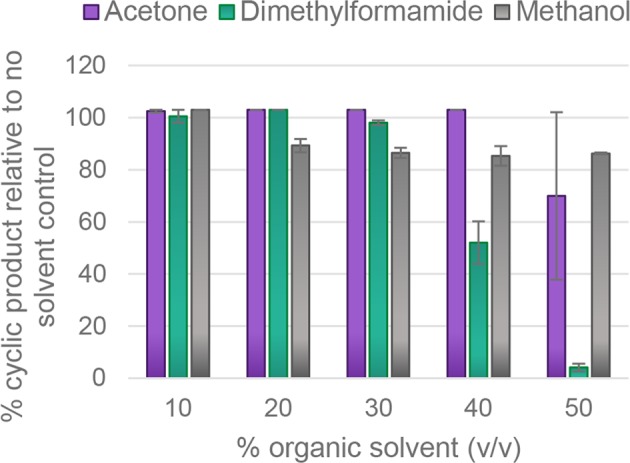
*OaAEP4 is tolerant to acetone*, *N-N-dimethylformamide and methanol*. Recombinant OaAEP4 (0.104 µM) was incubated with the R1 peptide (**K**L–NGL variant) (280 µM) at pH 5 for 60 min in the presence of increasing concentrations of organic solvents. The assays were conducted in activity buffer (50 mM sodium acetate buffer, pH 5.0, 0.5 mM NaCl, 1 mM EDTA, 0.5 mM TCEP) at room temperature. The products were assessed by MALDI-MS. The proportion of cyclic product was determined relative to all peaks attributed to the processed or unprocessed target peptide. Reactions were stopped by heating at 70 °C for 5 min. The average of two technical replicates is shown and error bars report the range.

Some target peptides are only soluble in organic solvents and enzymes that can tolerate organic co-solvents are therefore highly desirable. To examine whether recombinant AEP ligases could also cyclise peptides in different co-solvents, enzyme activity was examined in 0–50% (v/v) N,N-dimethylformamide (DMF), acetone and methanol (Figs 4 and S6). OaAEP4 was the best performing enzyme in the presence of organic solvents and could maintain yields close to 100% cyclic product (as judged by MALDI MS analysis) in up to 50% methanol, 40% acetone or 30% DMF (Fig. 4).

### Cyclisation of an intrinsically disordered protein, MSP2

**Figure 5:**
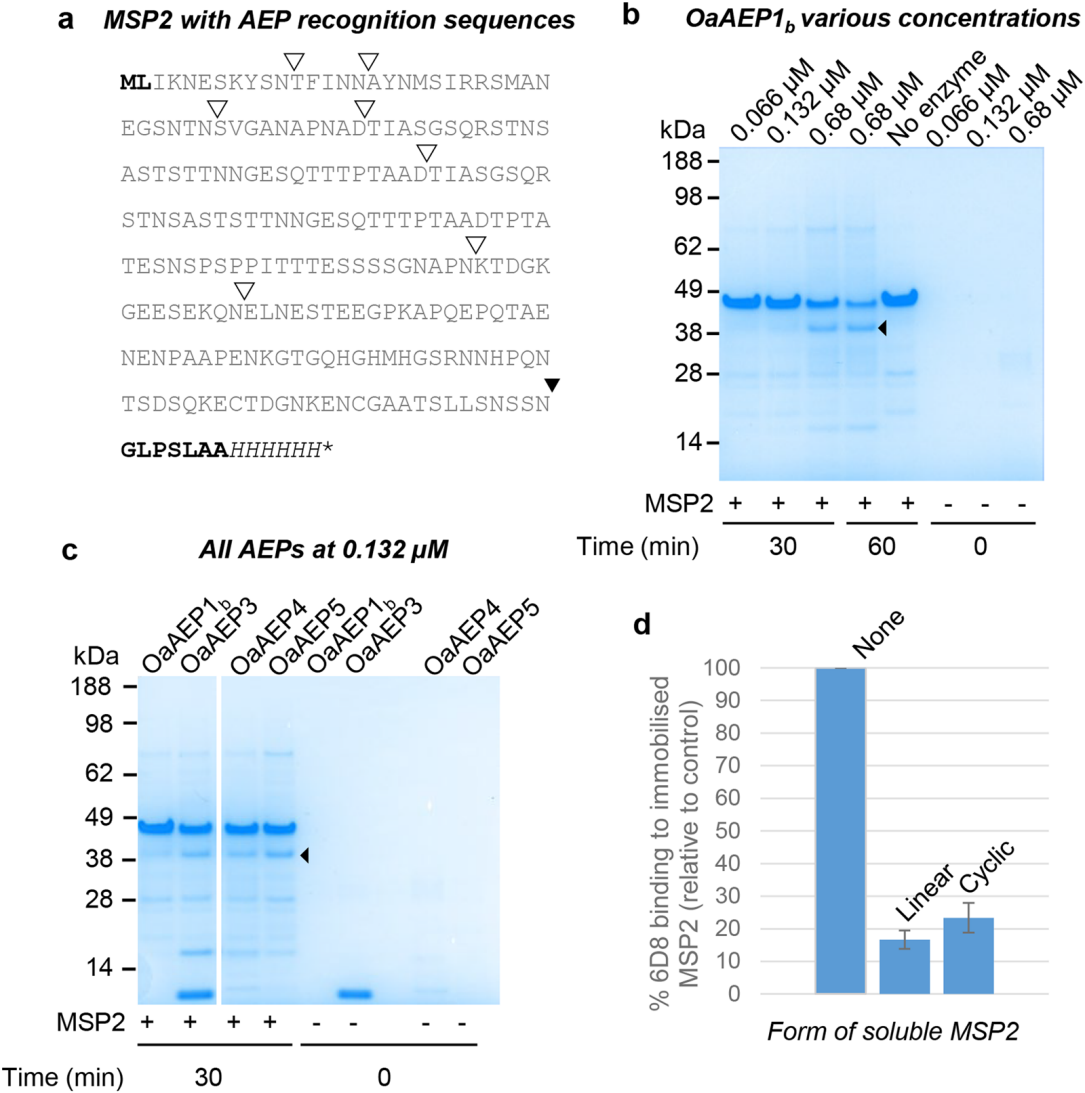
*An intrinsically disordered protein with AEP recognition residues can be backbone cyclised*. (**a**) Protein sequence of the MSP2_AEP_ produced recombinantly. The N-terminal recognition motif (NTRM; ML) and C-terminal recognition motif (CTRM; GLPSLAA) are shown in bold. The NTRM was altered to ML to accommodate the initiating methionine. ▾Denotes the target AEP processing site which is followed by the CTRM and a C-terminal 6xHis tag (shown in italics). ∇Denotes potential off-target AEP processing sites, as determined by similarity to the native minimal CTRM, N/GL. (**b**) SDS PAGE analysis of MSP2 (3 µg) incubated with and without recombinant OaAEP1_b_. The assays were conducted in activity buffer without reducing agent (50 mM sodium acetate buffer, pH 5.0, 50 mM NaCl, 1 mM EDTA). The dominant processing product indicated by ◂ was purified by size exclusion chromatography and confirmed to be the cyclic product by ESI-MS. MSP2 only and enzyme only controls are also shown. A single representative experiment of two technical replicates is shown. The gel has been cropped and an uncropped image is shown in Supplementary Fig. S8. (**c**) SDS PAGE analysis of MSP2 (3 µg) incubated with recombinant OaAEP1_b_, OaAEP3, OaAEP4 and OaAEP5 at 0.132 µM for 30 min. Enzyme only controls are also shown. ◂Denotes the product assigned as backbone cyclised MSP2. The grouping of different parts of the same gel is indicated by the white space. A single representative experiment of two technical replicates is shown. The gel has been cropped and an uncropped image is shown in Supplementary Fig. S8. (**d**) An anti-MSP2 monoclonal antibody 6D8 was allowed to interact with immobilised MSP2 in the presence or absence of soluble MSP2 (linear or cyclic). Both cyclic and linear soluble MSP2 proteins inhibited the interaction of MAb 6D8 with immobilised MSP2. A control with no soluble MSP2 added was set as 100% 6D8 binding, and the impact of the addition of linear or cyclic MSP2 is reported relative to this. The average of two technical replicates is shown and error bars report the range.

Intrinsically disordered proteins lack a well-defined structure and are inherently flexible. To assess whether the cyclisation ability of the suite of AEPs could extend to an intrinsically disordered protein, we used the malarial vaccine candidate, *Plasmodium falciparum* merozoite surface protein 2 (MSP2)^26,27^. The intrinsically disordered nature of recombinant MSP2 has been well-established using extensive NMR studies, analytical ultracentrifugation, dynamic light scattering and analytical size exclusion chromatography (SEC)^26,27,37^. In this study, full-length MSP2 carrying AEP recognition motifs was produced in *E*. *coli* (MSP2_AEP_, Fig. 5a). MSP2_AEP_ contained a single extra N-terminal residue (Leu in position two) and seven extra C-terminal residues (Gly-Leu-Pro-Ser-Leu-Ala-Ala) compared to the recombinant MSP2 used in vaccine trials. Consistent with disorder, MSP2_AEP_ eluted as a much larger protein on SEC (Superdex 75 10/300) and its retention time was identical to the previously characterised recombinant MSP2 lacking AEP recognition motifs^26^ (Supplementary Fig. S7).

Using standard protein expression protocols, the first residue of a recombinant protein is the initiating Met, restricting the nature of the N-terminal recognition motif. However, because these recombinant AEP ligases do not impose strict requirements at this position^1^ (Fig. 2a), it was anticipated that an N-terminal motif of Met-Leu would be well-tolerated. The C-terminal motif was extended to incorporate the entire C-terminal propeptide of the native OaAEP1_b_ substrate, kB1 (Gly-Leu-Pro-Ser-Leu-Ala-Ala)^1^, followed by a 6xHis tag. Since residues downstream of the P1 Asn are not present in the final product (Fig. 2b) the C-terminal peptide does not impact on the foreign residue footprint and allows cyclisation and removal of the purification tag in a single step.

When analysed by SDS-PAGE, MSP2_AEP_ runs as a much larger protein than the 25 kDa mass predicted from its amino acid sequence (Figs 5b, S8). This discrepancy is consistent with previous reports and is assumed to be due to its extreme hydrophilicity resulting in low binding of SDS^26^. Recombinant MSP2 has previously been confirmed to be monomeric by analytical ultracentrifugation^26^. Incubation of MSP2_AEP_ with recombinant OaAEP1_b_ resulted in a dominant processing product with greater electrophoretic mobility than the precursor protein, consistent with backbone cyclisation (Figs 5b, S8). Other minor processing products were also evident, probably a result of off-target processing of internal Asn/Asp residues and inter-molecular ligation. Analysis of the MSP2_AEP_ sequence identified seven Asx residues most likely to be targeted during the short timeframe of the assay because they are followed by a hydrophobic P2′ residue (Fig. 5a), as is preferred by OaAEP ligases (Fig. 2a). The larger species are likely to be products of inter-molecular ligation whereas the smaller species may be cyclic products generated from these internal potential AEP-cleavage sites. Consistent with the occurrence of at least some off-target processing, increasing the incubation time (30 min to 60 min) did not increase the amount of the putative cyclic product, despite the decrease in precursor levels (Figs 5b, S8).

To confirm that backbone cyclisation had occurred, the putative cyclic product was gel extracted and subjected to trypsin digestion followed by tandem MS analysis. Multiple peptides spanning the cyclisation point were identified, confirming backbone ligation had occurred (Supplementary Fig. S9). The putative cyclic product was purified using size exclusion chromatography (Superdex 200, 16/600) and the mass of the intact product was consistent with predominantly backbone-cyclised MSP2_AEP_, as determined by electrospray ionisation (ESI) MS (observed average mass, 23,115.0 Da; expected average mass, 23,115.0 Da) (Supplementary Fig. S10).

Secondary structure analysis by circular dichroism (CD) revealed that cyclic MSP2_AEP_ remained largely unstructured, as evidenced by a single minimum at 198 nm and weak negative ellipticity at 215–235 nm, which is indicative of a small amount of secondary structure (Supplementary Fig. S11). This is consistent with the characteristics reported previously for linear FC27 MSP2^27^, indicating the backbone cyclisation had not induced any major change in secondary structure. The purified cyclic product retained reactivity with an anti-MSP2 monoclonal antibody (MAb 6D8)^28^ in solution, as judged by an inhibition ELISA (Fig. 5d). MAb 6D8 is poorly reactive with native (parasite-derived) MSP2, suggesting that cyclic MSP2_AEP_ may not be antigenically similar to the native antigen.

To generate cyclic MSP2_AEP_, a relatively high amount of rOaAEP1_b_ was required (0.03 molar equivalent). Given that OaAEP1_b_ had less favourable kinetics than the other enzymes tested, including a far higher *K*_m_ (Table 1) and less effective conversion of two peptide substrates to their cyclic products (Fig. 1), we predicted that the other recombinant AEPs would be more effective at cyclising MSP2. Indeed, when OaAEP 3–5 were tested, 5-fold less enzyme was sufficient for similar apparent levels of production of cyclic MSP2_AEP_ (Figs 5c, S8).

## Discussion

This study reports the production and characterisation of three new plant-derived AEP ligases with superior kinetics to the previously described OaAEP1_b_. These AEPs can work as dominant ligases at both acidic and neutral pH, leading to their classification as “canonical AEP ligases”, distinguishing them from AEPs that shift their activity preferences with pH. The cyclisation capabilities of these canonical AEP ligases are maintained in a range of organic co-solvents and extend to a 25 kDa intrinsically disordered protein, the malarial antigen MSP2, highlighting their broad applicability as tools in peptide and protein engineering.

Some plant AEPs can change their activity preferences according to pH, working as proteases under acidic conditions (eg. pH 5) but with increased ligase activity emerging as the pH approaches neutrality (pH 6.5)^18–20^. However, the enzymes we report here are different because they continue to work preferentially as ligases at both acidic and neutral pH, at least on the substrate tested (Fig. 3). When the sequences of these two types of AEPs were compared at the ligase predictive residues^14^, only the canonical AEP ligases consistently presented residues associated with ligase activity (Supplementary Fig. S5). This raises the possibility that these sequence signatures specifically underpin canonical ligase activity, whilst other sequence combinations control pH-dependent ligase activity. Indeed, to carry out the sequence space analysis used to identify key residues, AEPs were typically classified as ligase type if they were effective at cyclising the prototypic cyclotide kalata B1 *in planta*. Given that expression *in planta* may require ligase function to be maintained at acidic (vacuolar) pH^14^, this could feasibly skew selection towards canonical AEP ligases, and away from pH-dependent ligases. The discovery and functional characterisation of more enzymes will be required to further clarify the molecular features of these different types of AEPs. It should also be noted that the enzymes described here are produced recombinantly in *E*. *coli* and are therefore not glycosylated. In their native environment, it is possible that these enzymes are glycosylated and may display different properties as a result.

The canonical AEP ligases reported here retained activity in a range of solvents. Excellent yields were achievable in up to 50% (v/v) acetone and methanol and 30% (v/v) DMF, depending on the enzyme (Figs 4, S6). The ability to carry out cyclisation in the presence of organic co-solvents makes this an accessible technique for highly hydrophobic peptides that are not soluble in aqueous solutions, further broadening the applications of AEP ligases. In traditional peptide bond hydrolysis by cysteine proteases, an acyl-enzyme thioester intermediate is formed, and nucleophilic attack of a water molecule is required to resolve this. However, during peptide cyclisation (or transpeptidation) the substrate’s N-terminal amine is postulated to function as a competing nucleophile, facilitating aminolysis of the reactive thioester intermediate^38^. Since water is not required for cyclisation, organic solvent tolerance will be dictated primarily by the stability of the enzyme within the solvent being tested. This could be investigated in follow up studies by monitoring enzyme unfolding in the presence of increasing concentrations of organic co-solvent, for example by circular dichroism.

Mechanistically, the way in which AEP ligases favour aminolysis over hydrolysis is not well understood. Using the structure of the pH-dependent ligase AtAEPγ, it was recently proposed that the ligase activity of AEPs with hydrophobic S2′ binding pockets depends on the presence of a complementary hydrophobic substrate residue at the P2′ position to exclude water from the active site^18^. In the context of pH-dependent AEP ligases, this S2′/P2′ interaction was deemed critical for determining whether the enzyme would perform hydrolysis or ligation. Interestingly, the canonical AEP ligases studied here present identical residues within the corresponding S2′ binding pocket (Supplementary Fig. S5) but continue to act as dominant ligases when a charged residue is supplied at the substrate P2′ position, albeit with much slower reaction kinetics (Fig. 2c). This suggests that their ligase activity *per se* is not intrinsically dependent on the nature of the S2′/P2′ interaction and that other molecular features probably underpin the preference of canonical AEP ligases for amines as the nucleophile instead of water. This is consistent with our previous finding that, in the context of OaAEP1_b_, the P2′ Leu residue that is highly conserved in native cyclotide substrates is important only for promoting appropriate enzyme-substrate interaction and not for excluding water from the active site to prevent premature hydrolysis^1^. Indeed, the active site of OaAEP1_b_ is accessible to water, as evidenced by the slow hydrolysis of a modified cyclotide substrate lacking a free amine at the N-terminus, suggesting that OaAEP1_b_ inherently favours transpeptidation^1^. This is consistent with a recent report that PatG intrinsically favours free amines as the nucleophile rather than relying on the hydrophobic exclusion of water via its “capping helices”^39^.

Accurate comparison of the kinetics of different enzymes requires careful quantitation of the active enzyme component by active site titration that was not carried out in previous studies describing AEPs. Here, active site titrated enzymes were used to more reliably compare enzyme activity (Table 1). Despite procedural differences, the kinetic parameters of OaAEP1_b_ determined on the R1_IQF_ peptide substrate (*k*_cat_ 0.99 s^−1^; *K*_m_ 146 µM; *k*_cat_*/K*_m_ 6774 M^−1^s^−1^) were in a similar range to that reported previously on the native kalata B1 substrate (*k*_cat_ 0.53 s^−1^; *K*_m_ 212 µM; *k*_cat_*/K*_m_ 2500 M^−1^s^−1^)^1^. However, the turnover rates reported here are approximately 20-fold higher than that in another report^12^. This discrepancy could be explained by factors such as the different substrates profiled, different enzyme quantitation methods or an over-estimation of the maximum enzyme velocity because of the high apparent *K*_m_ of OaAEP1_b_ for the specific substrate used.

Interestingly, the previously uncharacterised AEPs, OaAEP3, OaAEP4 and OaAEP5, had far lower *K*_m_ values than OaAEP1_b_ and performed better in cyclisation assays despite lower apparent turnover rates, making them kinetically superior. For these enzymes, the maximum turnover rate could be achieved without requiring high substrate concentrations resulting in very high catalytic efficiencies (*k*_cat_*/K*_m_ up to 329,982 M^−1^s^−1^). Most importantly, low amounts of enzyme (as little as 0.0001 nmol of enzyme per nmol of substrate) with a 2 h incubation time, (Fig. 3a, OaAEP4 at pH 4.2) could achieve cyclic peptide yields of close to 100%, as judged by MALDI MS. This compares favourably to values reported for butelase-1 (approx. 0.0005 molar equivalents)^5^ and omniligase (<0.0003 molar equivalents), an engineered derivative of subtiligase^40,41^.

In addition to short peptides, AEPs can also cyclise proteins^6^, but until now this application has been limited to globular proteins where the N- and C-termini are brought into close proximity by the protein fold. Here, we extend the capabilities of AEP ligases by demonstrating the backbone-cyclisation of an intrinsically disordered protein, the ~25 kDa malarial vaccine candidate MSP2 (Figs 5 and S7–11).

MSP2 was selected for cyclisation because it was predicted that cyclic MSP2 could more closely resemble native MSP2 displayed at the surface of the malaria parasite leading to improved performance as a vaccine. However, this hypothesis was not supported by our data for two reasons. Firstly, MAb 6D8 bound to the cyclised recombinant protein (Fig. 5d). This antibody is known to be poorly reactive with native MSP2^28^, suggesting that an epitope that is cryptic in native MSP2 is available in the cyclic protein. Secondly, the cyclic product was predominantly disordered (Supplementary Fig. S11), consistent with the unstructured nature of linear recombinant MSP2^27^. However, to definitively determine if cyclic MSP2 offers an advantage as a vaccine candidate, studies comparing the fine specificity of antibodies generated by cyclic versus linear recombinant MSP2 would be required.

The lack of a stable three-dimensional structure across the entire length of intrinsically disordered proteins presents a new challenge for enzymatic cyclisation: off-target processing sites that might be inaccessible in a more globular protein may now be more readily available. When only those Asx residues that are followed by a hydrophobic residue in the P2′ position are considered, MSP2 presents seven potential off-target processing sites (Fig. 5a). This is likely to impact the achievable yield since, although the cyclic product is generally dominant, increased incubation time resulted in depletion of the precursor protein without a corresponding increase in cyclic product (Figs 5b, S8). Strategies to increase yield could include mutagenesis of key potential off-target sites. For example, P1 Asx to Glu mutagenesis or replacement of a hydrophobic P2′ residue with a charged residue such as His (refer to GL–NG**H** peptide in Fig. 2), however the feasibility of this will depend on the target protein.

This study describes three catalytically superior recombinant AEPs with broad scope in the cyclisation of not only peptides, but also a highly challenging intrinsically disordered protein. These canonical AEP ligases retain their ligase activity at both acidic and neutral pH on the substrate tested, distinguishing them from those that are pH-dependent. The low molar ratios of enzyme required and short incubation times, together with a broad pH range of activity and tolerance to a range of commonly used organic solvents, will ensure these enzymes find wide application in biotechnology.

## Experimental Procedures

### Peptide substrates and inhibitors

Internally-quenched fluorescent (IQF) peptides containing an *o*-aminobenzoic acid (ABZ) group and a C-terminal 3-nitrotyrosine (Y[3NO_2]_) were synthesised by GL Biochem at >90% purity and were quantitated by amino acid analysis. These peptides were ABZ-STRNGLPS-Y-(3NO_2_) and GLPVFAEFLPLFSKFGSRMHILK(K-ABZ)STRNGLPS-Y(3NO_2_). The control fluorescent peptide ABZ-STRN was also synthesised by GL Biochem at >90% purity and quantitated by amino acid analysis. All IQF or fluorescent peptides were solubilised in 25% (v/v) acetonitrile:water. The caspase inhibitor Ac-YVAD-CMK (where Ac, acetyl; CMK, chloromethylketone) was supplied by Peptides International and solubilised in dimethyl sulfoxide (DMSO) to 10 mM. All R1 and IK8 peptide variants were supplied by GL Biochem at >85% purity, dissolved in ultrapure water prior to analysis and quantitated using the Direct Detect system (Milllipore) according to the manufacturer’s instructions.

### Recombinant expression and purification of *O*. *affinis* AEPs

The sequence of OaAEP3 was reported previously (Genbank accession code KR259379)^1^ and it was recently assigned as a ligase in planta^13^. OaAEP4 and OaAEP5 were two of the theoretical AEP transcripts identified from previous *O*. *affinis* transcriptomics^1^. These AEPs were recombinantly expressed, along with OaAEP1_b_ (Genbank accession code KR259379), and their protein sequences are listed in Supplementary Fig. S1. DNA encoding all AEPs (without the putative signalling domain) was inserted into the pHUE vector^42^. OaAEP4 and OaAEP5 sequences were codon optimised for expression in *Escherichia coli* whereas OaAEP1_b_ and OaAEP3 DNA sequences were as isolated from their native source.

OaAEP4 and OaAEP5 were expressed and purified as described previously^1^. Briefly, the expression of His6-ubiquitin-OaAEP fusion protein constructs was induced by isopropyl ß–D-1-thiogalactopyranoside (IPTG) when cells were in log phase. After ~20 hours, cells were harvested, lysed, and recombinant protein was captured by anion exchange. AEP-positive fractions were self-activated by incubation at pH 4.5 before a final cation exchange step to capture mature, active enzyme.

OaAEP1_b_ and OaAEP3 were expressed and purified using a previously described method^1^, with the following modifications. After cells were harvested, they were resuspended in non-denaturing lysis buffer (20 mM phosphate buffer, pH 8.0, 10 mM imidazole, 0.3 M NaCl) using 60 ml lysis buffer/L of culture. Lysis was achieved by bead beating in a GenoGrinder (SPEXSamplePrep) using 20 g of 100 μm silica beads per 20 mL lysate. DNase (bovine pancreas; 20 µg mL^−1^) and MgCl_2_ (20 mM) were then added to allow digestion of DNA. After a 30 min incubation, cellular debris was removed by centrifugation and the lysate was filtered through a 1 µm glass fibre filter prior to incubation with nickel-nitrilotriacetic acid (Ni-NTA) resin (2 mL of a 50% slurry per L of culture). Bound proteins were eluted with elution buffer (20 mM phosphate buffer, pH 8.0, 250 mM imidazole, 0.3 M NaCl) and AEP-positive fractions were pooled, concentrated and applied to a size exclusion column (Superdex 75 16/60, GE Healthcare). This enabled buffer exchange into activation buffer (50 mM sodium acetate, pH 4.0, 0.5 M NaCl). After addition of Tris(2-carboxyethyl)phosphine hydrochloride (TCEP; 0.5 mM) and ethylenediaminetetraacetic acid (EDTA; 1 mM), AEP-containing fractions were incubated for 4–5 h at 37 °C to facilitate self-maturation and active enzyme was captured by a final cation exchange step, as previously described. The total concentration of protein was estimated by BCA assay according to the manufacturer’s instructions and active site titration, as described in the next section.

### Active site titration of recombinant AEPs

The recombinant enzymes were active site titrated essentially as described in^34^. The enzyme preparation was diluted using activity buffer (50 mM sodium acetate buffer, pH 5.0, up to 50 mM NaCl, 1 mM EDTA, 0.5 mM TCEP). As reported in other AEP assays^1,2^, a reducing agent was included in the activity buffer to protect the active site cysteine from oxidation, however its inclusion was not essential for enzyme activity and it was omitted where indicated to avoid reduction of disulfide bonds in the target molecule. OaAEP1_b_ is reported to contain a single disulfide bond^12^ and this is likely for OaAEP3, 4 and 5 as well. It is not known if this disulfide remains in place under the assay conditions or, alternatively, if it is essential for activity. Serial dilutions (1:2) of the inhibitor Ac-YVAD-CMK were prepared in a black microtitre plate (Greiner Bio-One) using the activity buffer as diluent. The enzyme was added to the wells containing inhibitor Ac-YVAD-CMK and the volume in the relevant wells was made up to 90 µL with activity buffer. The final enzyme dilution was selected to ensure enough signal was generated without saturating the system. The plate was incubated for 1–5 h, depending on the enzyme, at room temperature prior to addition of the self-quenched substrate ABZ-STRNGLPS-Y(3NO_2_) (15 µM). Upon addition of the substrate the plate was read in kinetic mode for fluorescence on a SpectraMax M2 (Molecular Devices) using high sensitivity. For data acquisition, excitation and emission wavelengths of 320 and 420 nm were used respectively. Progress curves showing relative fluorescence units (RFU) plotted against time were generated. The initial rates (V_i_) were calculated during the linear portion of the progress curve. This initial rate was expressed relative to the initial rate of the no inhibitor control (V_0_). V_i_/V_0_ was then calculated and plotted against inhibitor concentration to create an inhibition curve. The titre of the enzyme active site was inferred from the x-axis intercept of the linear portion of this inhibition curve assuming a 1:1 interaction between enzyme and inhibitor. The concentrations of the enzyme stocks were thus calculated accounting for the dilution factors used in the relevant assays.

### Cyclisation of linear target peptides

Linear target peptides (280 µM) were incubated with the appropriate AEP in activity buffer. AEP concentrations are as indicated in the figure legends. The reaction was allowed to proceed for 10–60 min at room temperature after which time TFA was added to 0.1% (v/v). Where indicated, the enzyme was deactivated prior to this by incubation at 70 °C for 5 min. To profile AEP activity at different pH values the activity buffer remained the same except the following buffer systems were used: 50 mM citrate (pH 3.4); 50 mM sodium acetate (pH 4.1 and 5.1); 50 mM phosphate (pH 6.0, 6.9, and 8.1); 50 mM carbonate/bicarbonate (pH 9.4, 10.0). The pH was measured again after dilution with the appropriate volume of enzyme storage buffer and the final pH values indicated reflect this measurement.

The reaction mixture (5–20 µL) was de-salted using C18 zip tips (Millipore) and eluted in 4 µL 75% (v/v) acetonitrile, 0.1% (v/v) trifluoroacetic acid (TFA). When testing enzyme tolerance to organic solvents (0–50%[v/v] acetone, dimethylformamide [DMF] or methanol), samples were incubated for 1 h at room temperature in a 20 µl reaction volume, incubated at 70 °C for 5 min, then freeze dried. Lyophilised samples were then resuspended in 10 µl 0.1% (v/v) TFA in ultrapure water before being de-salted as described above.

Cyclisation of linear target peptides was monitored by matrix-assisted laser desorption/ionisation MS (MALDI MS). A saturated MALDI matrix solution (α-cyano-4-hyroxycinnamic acid, CHCA) prepared in 95% (v/v) acetonitrile, 0.1% (v/v) TFA was diluted 1:22 such that the final matrix solution comprised 90% (v/v) acetonitrile, 0.1% (v/v) TFA and 1 mM NH_4_H_2_PO_4_. De-salted samples were mixed 1:4 with the MALDI matrix, spotted onto a MALDI plate and analyzed by an Ultraflex III TOF/TOF (Bruker) in positive reflector mode.

For relative quantification of peptide products and precursors, the sum of the integrated areas of the peaks assigned to each peptide were determined in FlexAnalysis (Bruker). The percentage of cyclic peptide, relative to remaining precursor or any observed side products within the sample could then be calculated^13,14,17^.

### Real-time cyclisation kinetics

To assay activity of recombinant AEPs against the cyclisable IQF peptide, substrate and enzyme were diluted as appropriate in activity buffer (50 mM sodium acetate, 50 mM NaCl, 1 mM EDTA, 0.5 mM TCEP, pH 5). The change in fluorescence intensity over time was monitored on a SpectraMax M2 (Molecular Devices) using excitation/emission wavelengths of 320/420 nm. To determine real-time cyclisation kinetics, each substrate was assayed at a range of concentrations between 0.625–80 µM in a total volume of 100–200 µL. The concentration of active enzyme used was «substrate concentration. Relative fluorescence intensity (RFU) was converted to amount of product by comparison to a standard curve of the fluorescent peptide ABZ-STRN. At each substrate concentration, initial velocities were calculated from the linear portion of the progress curve. *K*_m_ and *V*_max_ were estimated using the Michaelis-Menten equation and the curve-fitting program GraphPad Prism 7 (GraphPad Software, San Diego).

At high concentrations of IQF peptides, high relative concentrations of the quenching group can impede detection of the signal from the fluorescent donor even after substrate hydrolysis^43^. This phenomenon is called the inner filter effect. This was accounted for using previously determined correction factors for the same donor/quencher pair^1^. The corrected signal for each data point was then converted to amount of product by comparison to a standard curve of the fluorescent peptide ABZ-STRN. A correction factor was only applied to substrate concentrations of 2.5 µM and above.

### Recombinant expression of linear MSP2

Full-length MSP2 (FC27 allelic form; GenBank Accession number JN248384)^44^ and MSP2 (FC27 allelic form) with additional AEP-recognition residues (MSP2_AEP_, Fig. 2b) and a hexa-his tag were produced recombinantly using a similar method to that described previously^27^. Briefly, the MSP2 DNA sequence was inserted into the pET22b vector and introduced into BL21(DE3) *E*. *coli* cells. Cells were grown to log phase (OD_600_ ~0.6) and expression was induced (5 h) by the addition of IPTG (1 mM). The culture was centrifuged and the cell pellet was resuspended in lysis buffer (20 mM Tris-HCl pH 8, 50 mM NaCl), boiled for 10 min then chilled for 20 min. This resuspension was then centrifuged and the supernatant passed over a Ni-NTA resin. MSP2-positive fractions were pooled and dialysed into 10 mM acetic acid. Prior to cyclisation assays the protein was lyophilised, resuspended in ultrapure water, and the concentration was determined using a Direct Detect system (Millipore). The retention volume of recombinant FC27 MSP2_AEP_ was compared to recombinant FC27 MSP2^26^ on a Superdex 75 10/300 size exclusion column (GE Healthcare) equilibrated in phosphate buffered saline (PBS) at a flow rate of 0.5 ml min^−1^.

### Preparation and analysis of backbone cyclised MSP2_AEP_

Full-length MSP2 (0.55 mg mL^−1^; 22 µM) was incubated with AEPs (0.066–0.68 µM) in non-reducing activity buffer (50 mM sodium acetate, 50 mM NaCl, 1 mM EDTA, pH 5) for 30–60 min. The reaction was monitored by SDS PAGE followed by Coomassie blue staining. To confirm the presence of cyclic product, the processing product was gel extracted and subjected to trypsin digest followed by tandem MS/MS analysis using an Orbitrap Elite (Thermo Scientific). The resulting spectra were analysed using PEAKS software (version 8.5; Bioinformatics Solutions, Inc.).

For purification of the cyclic product, TFA was added to a final concentration of 0.1% and the reaction mix was loaded onto a Superdex 200 16/60 size exclusion column (GE Healthcare) equilibrated in PBS at a flow rate of 0.5 ml min^−1^. Fractions positive for cyclic product by SDS PAGE followed by Coomassie blue staining were pooled and analysed further by ESI MS. Fractions were desalted using C18 zip tips (Millipore) according to the manufacturer’s instructions and eluted in 4 µL 75% (v/v) methanol, 1% (v/v) formic acid. 96 µl of 50% (v/v) methanol, 1% (v/v) formic acid was added to the desalted sample. The sample was then injected into a MicroTOF Q (Bruker) and data was collected in positive ionisation mode. The mass was determined by charge deconvolution using the Compass DataAnalysis program (Bruker).

### Circular dichroism spectroscopy of backbone cyclised MSP2_AEP_

CD spectra of cyclic MSP2_AEP_ were obtained on an Aviv 420 CD spectrophotometer over the wavelength range of 195–260 nm at a temperature of 25 °C. Cyclic MSP2_AEP_ was purified by size exclusion chromatography, dialysed into 10 mM sodium acetate pH 6.2 and the concentration adjusted to 0.2 mg mL^−1^. Data were collected using a step size of 1 nm, a slit bandwidth of 1.0 nm and a signal averaging time of 4.0 s in a 1 mm path length quartz cuvette (Hellma). CD spectra were fitted using a database comprised of 48 proteins (SDP48) and the CONTINLL algorithm using CDPro software.

### Inhibition ELISAs

Recombinant MSP2_AEP_ (Fig. 5a) was diluted to 2 μg mL^−1^ in PBS and immobilized on a 96-well microtiter plate (Maxisorp; Nunc) by overnight incubation at 4 °C. Unbound protein was removed by washing with PBS 0.05% Tween20 (PBST) before the plate was blocked with 5% skim milk powder in PBS. MAb 6D8^28^ (0.08 µg mL^−1^) was diluted in PBS and incubated with or without soluble MSP2_AEP_ (linear or backbone cyclised; 1.5 µg mL^−1^) for 1 h (room temperature) before it was applied to the microtiter plate. Following 1 h of incubation, unbound antibodies were removed by washing with PBST. An anti-mouse peroxidase-conjugated secondary antibody (1:1000; Pierce) was diluted in 5% skim milk powder in PBS and added to the wells. After 1 h of incubation, excess conjugate was removed by washing. Binding was visualized by the addition of 100 μl/well of o-phenylenediamine (OPD) (Thermo Scientific) according to the manufacturer’s instructions. The reaction was stopped by the addition of 50 µl/well of 1 M HCl and the absorbance was read at 490 nm.

## Supplementary information

Supplementary information

## Data Availability

All data generated or analysed during this study are included in this published article (and its Supplementary Information Files).
