# Supplementary material for: A suite of kinetically superior AEP ligases can cyclise an intrinsically disordered protein
Source: Sci Rep. 2019 Jul 25;9:10820. doi: 10.1038/s41598-019-47273-7 (PMC6658665; doi:10.1038/s41598-019-47273-7)
Supplement: Supplementary file 1 — Supplementary information [file 41598_2019_47273_MOESM1_ESM.pdf]

A suite of kinetically superior AEP ligases can cyclise an intrinsically disordered protein

Karen S. Harris[1], Rosemary F. Guarino[1], Ravindu S. Dissanayake[1], Pedro Quimbar[1], Owen C. McCorkelle[1], Simon Poon[1], Quentin Kaas[2], Thomas Durek[2], Edward K. Gilding[2], Mark A. Jackson[2], David J. Craik[2], Nicole L. van der Weerden[1], Robin F. Anders[1], Marilyn A. Anderson[1*]

[1]La Trobe Institute for Molecular Science, Melbourne, Vic. 3086, Australia, [2]Institute for Molecular Bioscience, The University of Queensland, Brisbane, QLD 4072, Australia.

[*]To whom correspondence should be addressed, Tel: +61 3 9479 1255, E-mail: m.anderson@latrobe.edu.au

Supplementary Figures S1-S11, Tables S1-S2

Table S1. *Percent identity matrix of OaAEP1$_b$, OaAEP3, OaAEP4 and OaAEP5, as determined by Clustal Omega.*

	OaAEP1b	OaAEP3	OaAEP4	OaAEP5
**OaAEP1b**	100	81.4	81.84	82.24
**OaAEP3**	81.4	100	94.2	94.07
**OaAEP4**	81.84	94.2	100	88.2
**OaAEP5**	82.24	94.07	88.2	100

```
                                       ↓                            ▼
OaAEP1b    1 MVRYLAGAVLLLV---VLSVAAAVSGARDGDYLHLPSEVSRFFRPQETNDDHGEDSVGTR  57
OaAEP4     1 MVRYPAGAVLL---LVVLSV-VA-VDGARDGYLKLPSEVSDFFRPRN--TNDGDDSVGTR  53
OaAEP3     1 MVRYLAGAFQVVLLVVILSDIAI-SEERTDGYLKLPTEVSRFFRTPEQSSDGGDDSIGTR  59
OaAEP5     1 MVRYLAGAFQVVLLVVILSDIAI-SEERTDGYLKLPTEVSRFFRTPEQSSDGGDDSIGTR  59

OaAEP1b   58 WAVLIAGSKGYANYRHQAGVCHAYQILKRGGLKDENIVVFMYDDIAYNESNPRPGVIINS 117
OaAEP4    54 WAVLLAGSNGYWNYRHQADLCHAYQILKRGGLKDENIVVFMYDDIAYNEENPRPGVIINS 113
OaAEP3    60 WAVLIAGSKGYDNYRHQADVCHAYQILKRGGLKDENIVVFMYDDIAYNESNPRPGVIINS 119
OaAEP5    60 WAVLIAGSKGYDNYRHQADVCHAYQILKRGGLKDENIVVFMYDDIAYNESNPRPGVIINS 119

                                                                      *
OaAEP1b  118 PHGSDVYAGVPKDYTGEEVNAKNFLAAILGNKSAITGGSGKVVDSGPNDHIFIYYTDHGA 177
OaAEP4   114 PHGSDVYAGVPKDYTGDEVNAKNFLAAILGNKSAITGGSGKVVDSGPNDHIFIYYTDHGA 173
OaAEP3   120 PHGSDVYAGVPKDYTGDEVNAKNFLAAILGNKSAITGGSGKVVDSGPNDHIFIYYTDHGA 179
OaAEP5   120 PHGSDVYAGVPKDYTGEEVNAKNFLAAILGNKSAITGGSGKVVDSGPNDHIFIYYTDHGA 179

                                                    *
OaAEP1b  178 AGVIGMPSKPYLYADELNDALKKKHASGTYKSLVFYLEACESGSMFEGILPEDLNIYALT 237
OaAEP4   174 PGVIGMPSKPYLYADELNDALRKKHASGTYKSMVFYLEACEAGSMFDGLLPDGLNIYALT 233
OaAEP3   180 PGVIGMPSKPYLYADELNDALRKKHASGTYKSMVFYLEACEAGSMFDGLLPDGLNIYALT 239
OaAEP5   180 AGVIGMPSKPYLYADELNDALKKKHASGTYKSLVFYLEACEAGSMFEGLLTDDLNIYALT 239

OaAEP1b  238 STNTTESSWCYYCPAQ-ENPPPPEYNVCLGDLFSVAWLEDSDVQNSWYETLNQQYHHVDK 296
OaAEP4   234 ASNTTEGSWCYYCPGQ-DAGPPPEYSVCLGDFFSIAWLEDSDVHNLRSETLNQQYHNVKN 292
OaAEP3   240 ASNTTEGSWCYYCPGQ-DAGPPPEYSVCLGDFFSIAWLEDSDVHNLRSETLNQQYHNVKN 298
OaAEP5   240 ASNATEGSCPYYCPGDLNYSPPPEYDVCLGDFFSIAWLEDSDVHNLRSETLNQQYHNVKN 299
                                            ▽▽    ▽ ▽            ▽ ▽
OaAEP1b  296 RISHASHATQYGNLKLGEEGLFVYMGSNPANDNYTSLDGNALTPSSIVVNQRDADLLHLW 356
OaAEP4   293 RISYASHATQYGDLKRGVEGLFLYLGSNPENDNYTFVDDNVVRPSSKAVNQRDADLVHFW 352
OaAEP3   299 RISYASHATQYGDLKRGVEGLFLYLGSNPENDNYTFVDDNVVRPSSKAVNQRDADLVHFW 358
OaAEP5   300 RISYASHATQYGDLKRGVEGLFLYLGSNPENDNYTFVDDNVVRPSSKAVNQRDADLVHFW 359

OaAEP1b  357 EKFRKAPEGSARKEVAQTQIFKAMSHRVHIDSSIKLIGKLLFGIEKCTEILNAVRPAGQP 416
OaAEP4   353 EKFRKAPEGSSKKEEAQKQILEAMSHRVHIDSSINLIGKLLFGIEKGHKILTAVRSAGHP 412
OaAEP3   359 EKFRKAPEGSSKKEEAQKQILEAMSHRVHIDSSINLIGKLLFGIEKGHKILTAVRSAGHP 418
OaAEP5   360 EKFRKAPEGSSKKEEAQKQILEAMSHRVHIDSSINLIGKLLFGIEKGHKILTAVRSAGHP 419

OaAEP1b  417 LVDDWACLRSLVGTFETHCGSLSEYGMRHTRTIANICNAGISEEQMAEAASQACASIP-- 474
OaAEP4   413 LVDDWACLRSLVRTFETHCGSLSQYGMKHTRTLANICNAGITEEQMAEAASQACVSIPSN 472
OaAEP3   419 LVDDWACLRSLVRTFETHCGSLSQYGMKHTRTLANICNAGITEEQMAEAASQACVSIPSN 478
OaAEP5   420 LVDDWACLRSLVGTFETHCGSLSEYGMRHTRTIANICNAGISEDQMKEAASQACASVPSN 479

OaAEP1b      ------------------
OaAEP4   473 PWSSHDGGFSA-------                                           483
OaAEP3   479 PWSSHDGGFSA-------                                           489
OaAEP5   480 SWSSLXKGFHARLAKIIA                                           497
```

Supplementary Figure S1: *Multiple alignment of AEPs using Clustal Omega.* Colour shows regions of identity. * denotes the catalytic dyad. ↓ denotes the putative signal peptide cleavage site and the first AEP residue of the recombinant protein. The final residue in each recombinantly expressed protein was Pro474 (OaAEP1$_b$), Ala489 (OaAEP3), Ala484 (OaAEP4) or Leu484 (OaAEP5). The recombinant AEPs are self-processed during incubation at low pH. ▼ and ▽show the N- and C-terminal self-processing sites respectively that were previously identified experimentally for recombinant OaAEP1$_b$ (1), defining the likely borders of the auto-activated proteins.

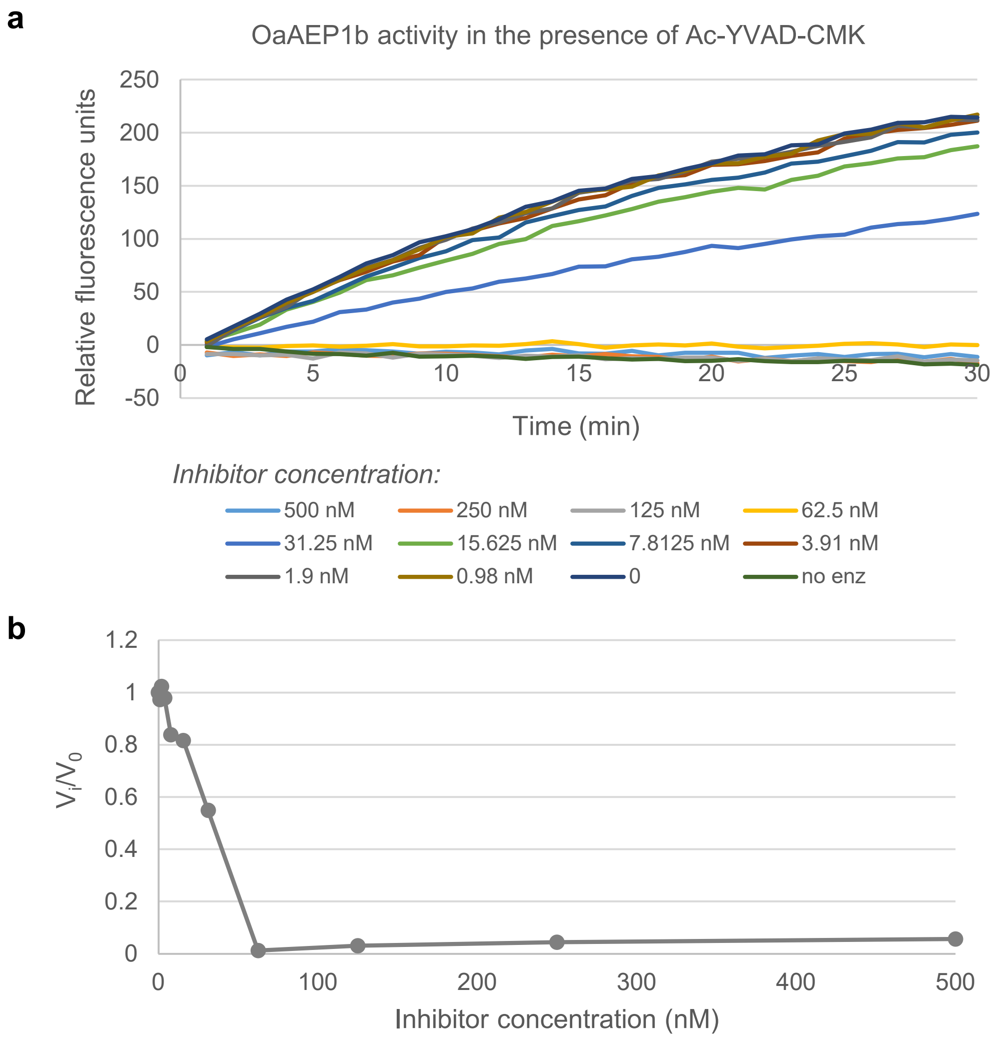

Supplementary Figure S2: *Active site titration for quantitation of active enzyme.* (a) Activity of OaAEP1_b against an IQF peptide (Abz-STRNGLPS-Y($3NO_2$)) in the presence of increasing concentrations of inhibitor. The assays were conducted in activity buffer (50 mM sodium acetate buffer, pH 5.0, up to 50 mM NaCl, 1 mM EDTA, 0.5 mM TCEP) at room temperature. OaAEP1_b is shown as a representative example. Other recombinant AEPs were quantitated in the same manner. (b) The initial rates (V_i) were calculated during the linear portion of the progress curve shown in (a). This initial rate was expressed relative to the initial rate of the no inhibitor control (V_0). V_i /V_0 was then plotted against inhibitor concentration. The x-intercept of the linear portion of the curve gives the concentration of active enzyme.

Table S2. *Summary data showing concentration of active enzyme determined by active site titration compared to concentrations based on BCA assay.*

Enzyme	Concentration (µM) (BCA assay)	Concentration (µM) (active site titration) (n≥3; ±sem)
OaAEP1b	31.25	6.17 ±0.135
OaAEP3	184	5.93 ±0.375
OaAEP4	9.375	0.33 ±0.04
OaAEP5	31.25	2.65 ±0.069

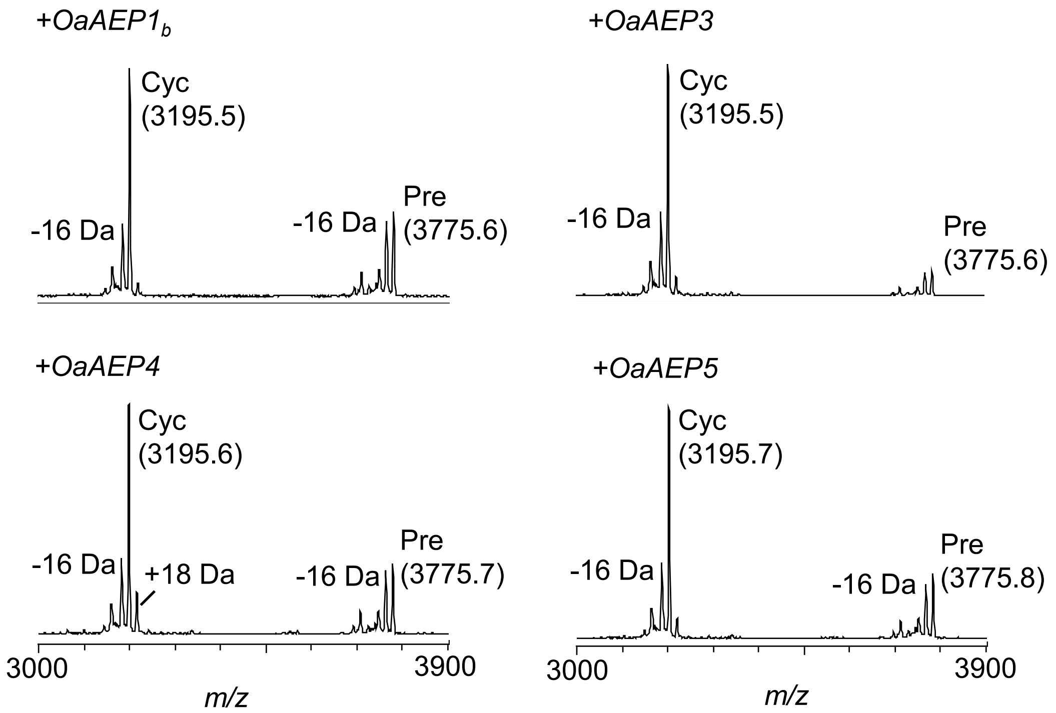

Supplementary Figure S3: *Products of enzymatic processing of the IQF peptide are cyclic.* Enzymes ($OaAEP1_b$, 60 nM; OaAEP3, 7.4 nM; OaAEP4, 6.6 nM; OaAEP5, 13 nM to reflect the concentrations used in the kinetic assays) were incubated with the R1 IQF peptide (30 µM) for 30 min and the products were analysed by MALDI MS. The assays were carried out in activity buffer (50 mM sodium acetate buffer, pH 5.0, 0.5 mM NaCl, 1 mM EDTA, 0.5 mM TCEP) at room temperature. The mass of the product observed is consistent with cyclised peptide. The observed average masses are listed (Da; $[M+H]^+$). The expected average mass of cyclic product (cyc) is 3194.64 Da. The expected average mass of precursor (pre) is 3775.2 Da. -16 Da peaks present in the precursor and product spectra are likely to represent a synthesis-derived modification. The mass of the product is consistent with cyclic peptide indicating that the real-time fluorescence assay is providing a read out of cyclic peptide production. A very small peak +18 Da from the cyclic product is observed, consistent with linear product. A single representative experiment of two technical replicates is shown.

Substrate

$R1_{IQF}$: GLPVFAEFLPLFSKFGSRMHIL(K-Abz)STRN↓GLPSY(3NO2)

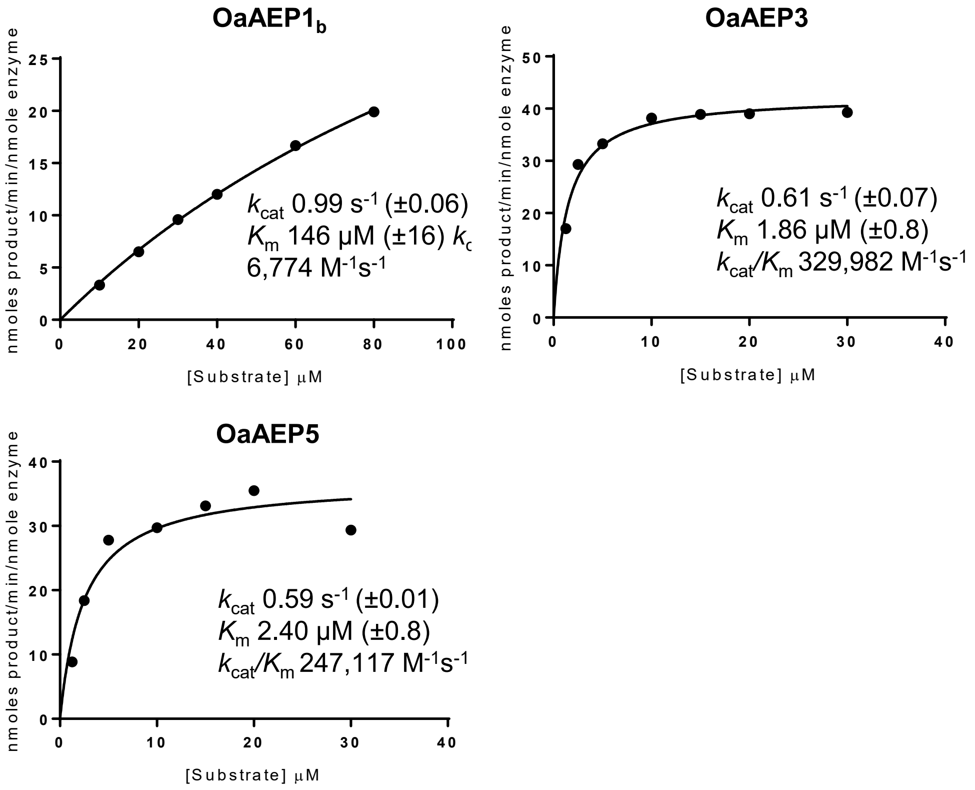

Supplementary Figure S4: *Michaelis-Menten kinetics of recombinant AEP ligases processing the $R1_{IQF}$ peptide.* A single representative Michaelis-Menten plot of at least three technical replicates is shown for $OaAEP1_b$, OaAEP3 and OaAEP5. The assays were carried out in activity buffer (50 mM sodium acetate buffer, pH 5.0, 50 mM NaCl, 1 mM EDTA, 0.5 mM TCEP) The average kinetic parameters from all experiments is reported. It is clear that $OaAEP1_b$ has a far higher K_m on this substrate that the other AEP enzymes. The very low K_m of OaAEP4 made determining accurate kinetic parameters challenging and estimates are reported in Table 1.

a S2’ binding pocket residues

	residue # (as in OaAEP1$_b$):	180	182	188	190
Canonical AEP ligases	OaAEP1$_b$:	V	G	Y	Y
	OaAEP3 :	V	G	Y	Y
	OaAEP4 :	V	G	Y	Y
	OaAEP5 :	V	G	Y	Y
pH-dependent ligases	AtLEGγ :	V	G	Y	Y
	RcAEP1 :	V	G	Y	Y
	HaAEP1 :	V	G	H	V
	AtLEGß :	V	G	H	Y

b Predictive residues for AEP ligase activity

	residue # (as in OaAEP1$_b$):	139	161	186	192	247	248	253	255	263	293	314	316
Canonical AEP ligases	OaAEP1$_b$:	K	D	K	D	C	Y	Q	N	V	HVDKR------ISH	E	G
	OaAEP3 :	K	D	K	D	C	Y	Q	A	V	NVKNR------ISY	V	G
	OaAEP4 :	K	D	K	D	C	Y	Q	A	V	NVKNR------ISY	V	G
	OaAEP5 :	K	D	K	D	P	Y	D	Y	V	NVKNR------ISY	V	G
pH-dependent ligases	AtLEGγ :	D	D	S	N	G	T	E	P	T	LVKRRTAPV-GYSY	K	N
	RcAEP1 :	K	D	L	M	G	T	M	P	T	SVKARTSNYNTYAA	A	K
	HaAEP1 :	D	D	E	K	G	T	T	P	T	KVKKRTSNSNTYNT	P	L
	AtLEGß :	A	A	T	A	G	T	M	P	T	TVKMRTSNYNTYAA	A	K
											MLA		

Supplementary Figure S5: *Comparison of AEP protein sequences at ligase predictive residues and the S2’ pocket.* (a) AEP sequences in the S2’ binding pocket, as identified in the AtLEGγ structure (Zauner et al, *JBC*. 2018). Residue numbering is as for OaAEP1$_b$. Hydrophobic residues replaced with a charged His are shown in purple. (b) AEP sequences at the 12 ligase predictive sites and the MLA (Jackson et al, *Nat. Comms.* 2018). Residues in blue are typically identified in AEP ligases. Residues in magenta are typically identified in AEP proteases. The MLA typically harbours a deletion (or increased hydrophobicity) in AEP ligases.

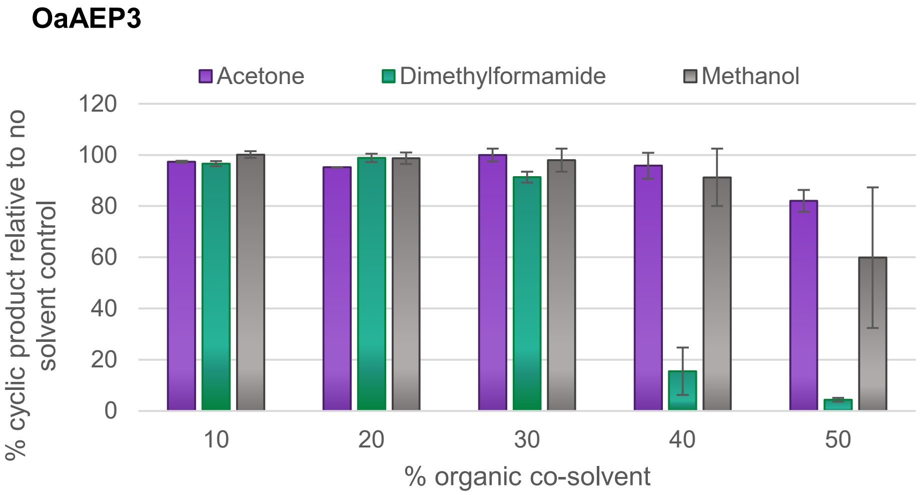

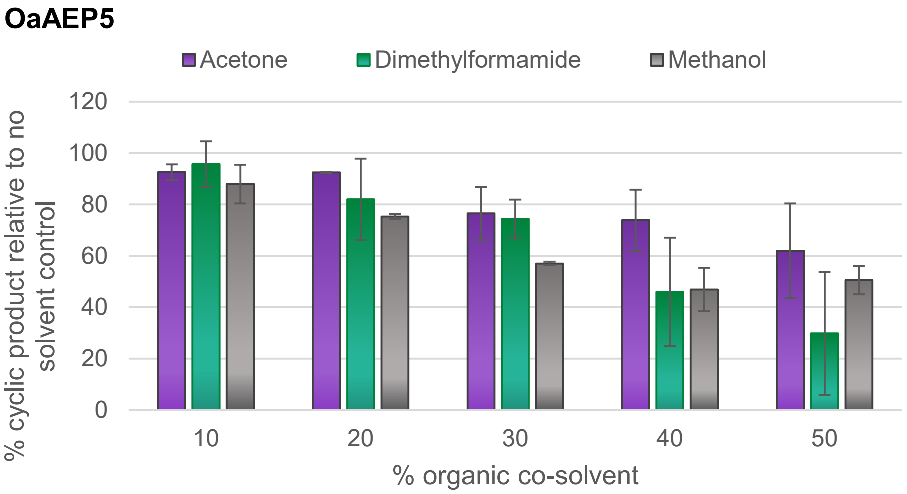

Supplementary Figure S6: *Solvent tolerance of OaAEP3 and OaAEP5.* Recombinant OaAEP3 (0.132 µM) and OaAEP5 (0.132 µM) were incubated with the R1 peptide (**K**L---NGL variant) (280 µM) at pH 5 for 60 min in the presence of increasing concentrations of solvents. The assays were carried out in activity buffer (50 mM sodium acetate buffer, pH 5.0, 0.5 mM NaCl, 1 mM EDTA, 0.5 mM TCEP) at room temperature. The products were assessed by MALDI-MS. The proportion of cyclic product was determined relative to all peaks attributed to the processed or unprocessed target peptide. Reactions were stopped by heating at 70 °C for 5 min. The average of two technical replicates is shown and error bars report the range.

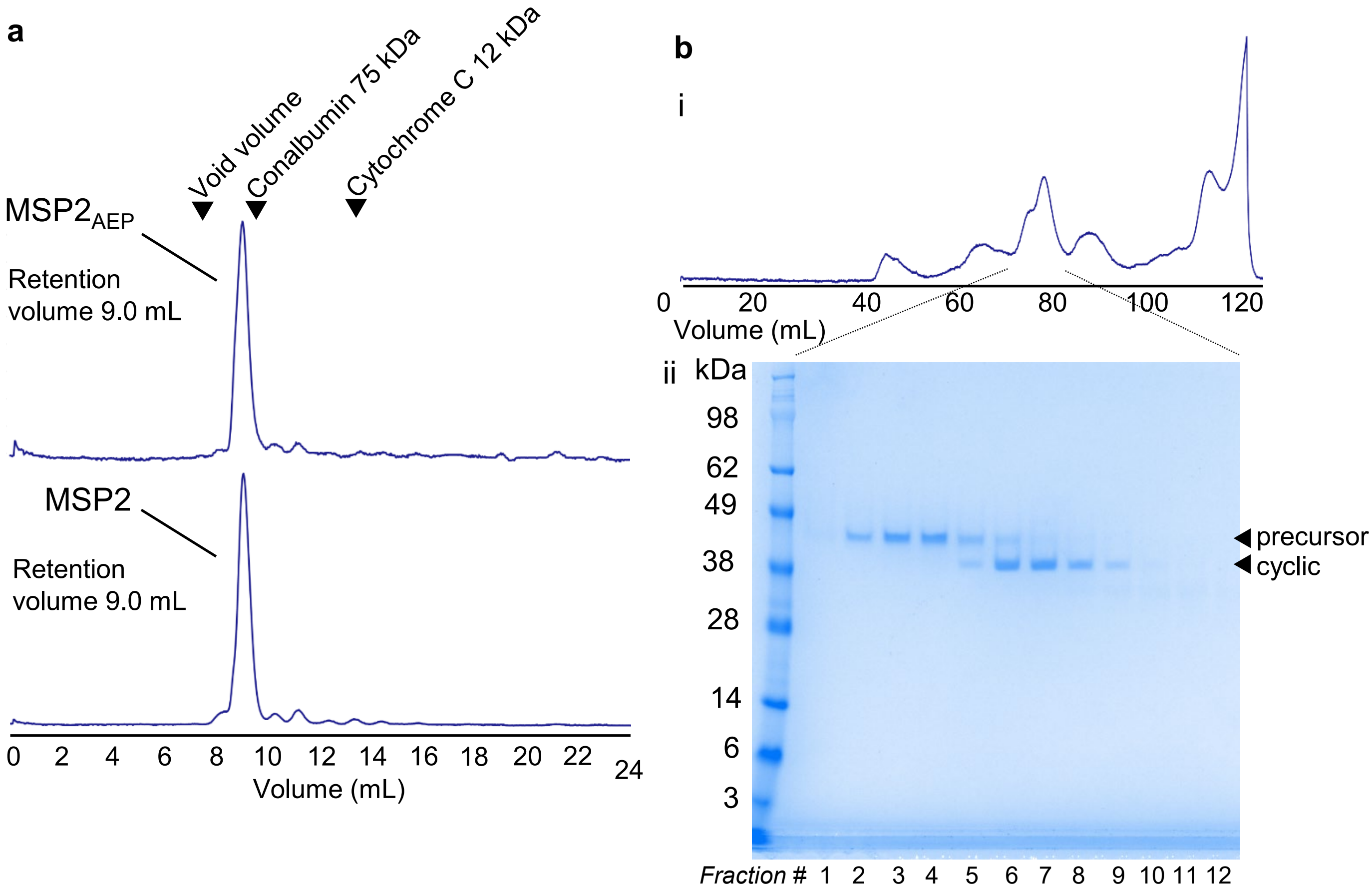

Supplementary Figure S7: *Size exclusion analysis of MSP2.* (a) $MSP2_{AEP}$ and recombinant MSP2 lacking the AEP recognition site were analysed by size exclusion (superdex 75 column). The two versions of recombinant MSP2 had identical retention volumes and their apparent Mw was much higher than their predicted Mw of 25 kDa. The experimentally determined retention volumes of the globular proteins conalbumin (75 kDa, 9.4 mL), and cytochrome C (12 kDa, 13.4 mL) are shown for comparison. The void volume was 7.6 mL, as determined using blue dextran. (b) $MSP2_{AEP}$ was processed by $OaAEP1_b$ (0.68 µM, 60 min) as in Fig. 5b and then applied to a superdex 200 size exclusion column to separate the precursor and cyclic product (i). Fractions were analysed by SDS-PAGE followed by Coomassie staining (ii). Fractions containing predominantly precursor or predominantly cyclic product were pooled.

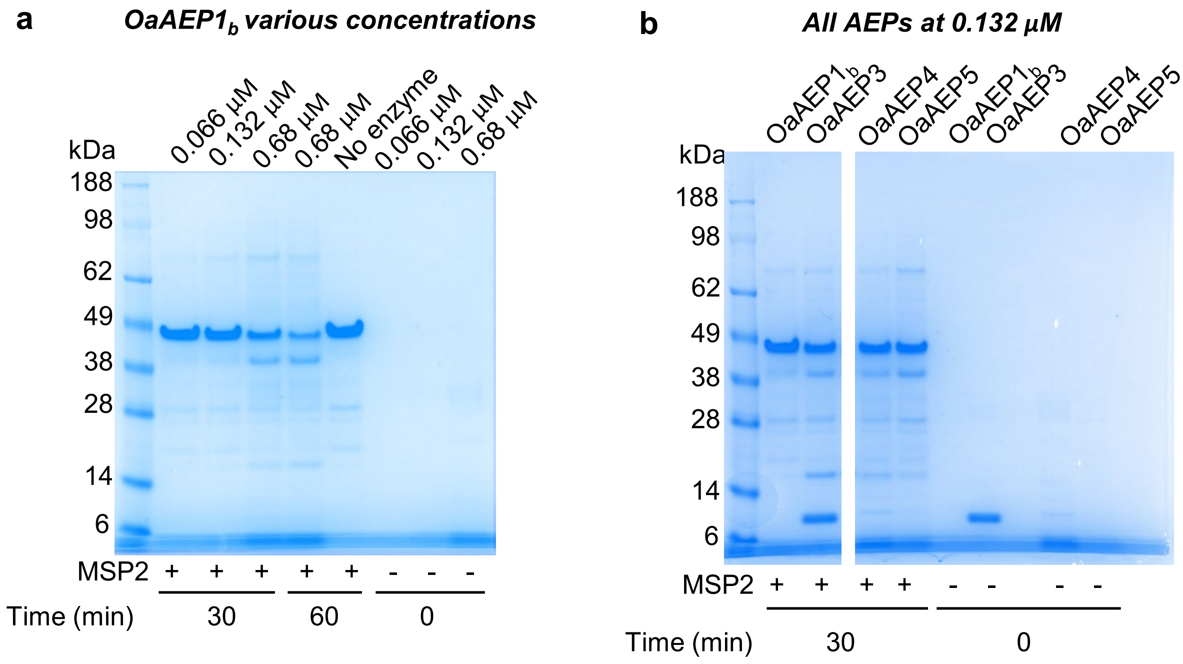

Supplementary Figure S8: *AEP-mediated processing of an intrinsically disordered protein.* (a) SDS PAGE analysis of MSP2 (3 µg) incubated with and without recombinant OaAEP1$_b$. The assays were conducted in activity buffer without reducing agent (50 mM sodium acetate buffer, pH 5.0, 50 mM NaCl, 1 mM EDTA). MSP2 only and enzyme only controls are also shown. A single representative experiment of two technical replicates is shown. (b) SDS PAGE analysis of MSP2 (3 µg) incubated with recombinant OaAEP1$_b$, OaAEP3, OaAEP4 and OaAEP5 at 0.132 µM for 30 min. Enzyme only controls are also shown. The grouping of different parts of the same gel is indicated by the white space. A single representative experiment of two technical replicates is shown. The gels are the same as those in Fig.5 b and c respectively and are displayed here in their entirety.

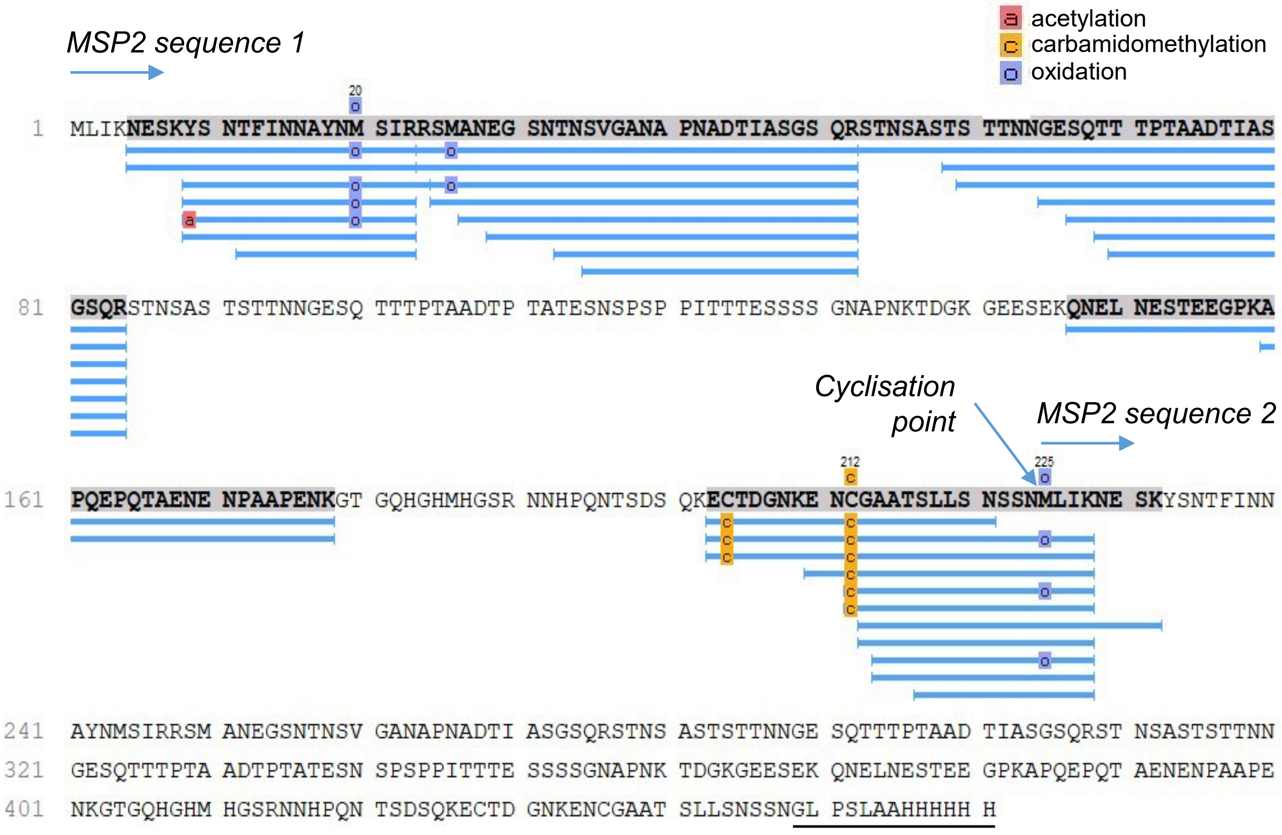

Supplementary Figure S9: *Sequence coverage of backbone cyclised MSP2$_{AEP}$ after tryptic digestion and tandem MS.* Two recombinant MSP2$_{AEP}$ sequences are listed consecutively. The first sequence lacks the leaving group released after enzyme processing (GLPSLAAHHHHHH, black underline in the second sequence) and is ligated to the N-terminus of a second MSP2 sequence This reproduces the sequence of backbone cyclised MSP2 across the cyclisation point (arrowed). The sequence coverage attained via tandem MS after tryptic digestion of the gel-extracted cyclic product is shown (blue underline). Multiple peptides spanning the cyclisation point were identified confirming the cyclic nature of the extracted protein.

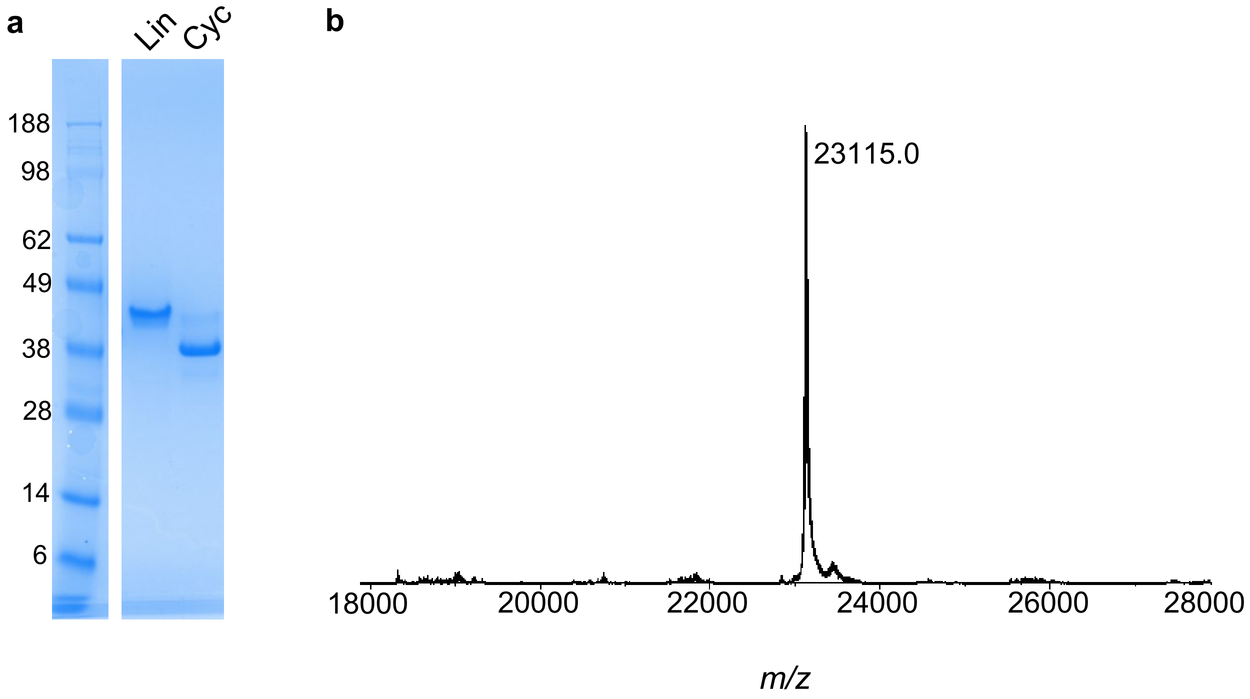

Supplementary Figure S10: *Cyclic MSP2$_{AEP}$ has greater electrophoretic mobility than linear MSP2$_{AEP}$.* (a) Linear precursor (Lin) and putatively cyclic (Cyc) versions of MSP2$_{AEP}$ were separated by size exclusion (superdex 200, 16/60) and analysed by SDS-PAGE. The grouping of different parts of the same gel is indicated by the white space. (b) The mass of the putative cyclic MSP2$_{AEP}$ was analysed by ESI-MS. The observed dominant average mass of 23,115.0 Da was consistent with the expected average mass of 23,115.0 Da of cyclic product with a single disulfide bond.

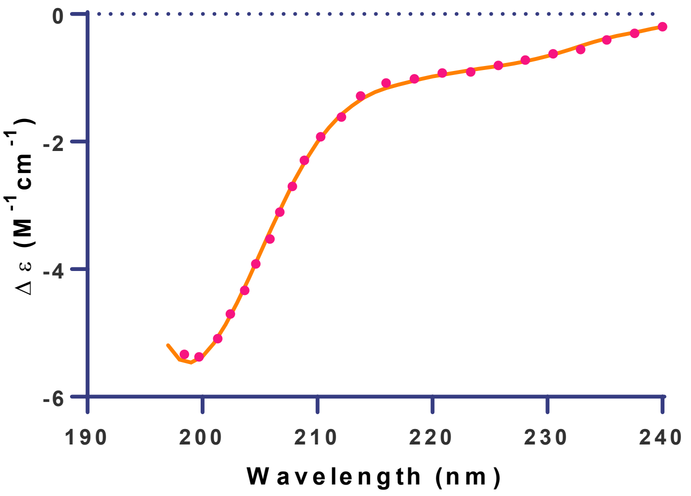

Supplementary Figure S11: *Circular dichroism spectroscopy of cyclic MSP2$_{AEP}$ in aqueous solution.* Difference in mean residue ellipticity, $\Delta\varepsilon$($M^{-1}cm^{-1}$), plotted as a function of wavelength (nm) for cyclic MSP2$_{AEP}$ solubilised in 10 mM sodium acetate pH 6.2 at a concentration of 0.2 mg mL^{-1}. The nonlinear least squares best fit using the CONTINLL algorithm with the SDP48 database is shown. The spectra is consistent with a predominantly disordered protein with a small portion of secondary structure.

References:

1. Harris, K. S., Durek, T., Kaas, Q., Poth, A. G., Gilding, E. K., Conlan, B. F., Saska, I., Daly, N. L., van der Weerden, N. L., Craik, D. J., and Anderson, M. A. (2015) Efficient backbone cyclization of linear peptides by a recombinant asparaginyl endopeptidase. *Nat. Commun.* **6**, 10199
